# Loop pathways are responsible for tuning the accumulation of C19- and C22-sterol intermediates in the *mycobacterial* phytosterol degradation pathway

**DOI:** 10.1186/s12934-022-02008-8

**Published:** 2023-01-30

**Authors:** Shikui Song, Jianxin He, Meng Gao, Yongqi Huang, Xiyao Cheng, Zhengding Su

**Affiliations:** 1 Key Laboratory of Industrial Fermentation (Ministry of Education), Cooperative Innovation Center of Industrial Fermentation (Ministry of Education and Hubei Province) and Hubei Key Laboratory of Industrial Microbiology, Hubei University of Technology, Wuhan, 430068 China; 2 School of Light Industry and Food Engineering, Guangxi University, No. 100, Daxuedong Road, Xixiangtang District, Nanning, 530004 Guangxi China; 3 Wuhan Amersino Biodevelop Inc., B1-Building, Biolake Park, Wuhan, 430075 Hubei China

**Keywords:** 1,4-androstadiene-3,17-dione (ADD), 22-hydroxy-23,24-bisnorchol-4-ene-3-one (BA), 3-ketosteroid-1,2-dehydrogenase (KstD), 3-ketosteroid-9α-hydroxylase (Ksh), 4-androstene-3,17-dione (4-AD), 9α-hydroxyl-4-androstene-3,17-dione (9OH-AD), 3-hydroxy-9,10-secoandrost-1,3,5(10)-triene-9,17-dione (HSA), Biotransformation, Cholesterol oxidases (Cho), Monooxygenase (Mon), Bioconversion, Phytosterols and *Mycobacteriumsp.*

## Abstract

4-Androstene-3,17-dione (4-AD) and 22-hydroxy-23,24-bisnorchol-4-ene-3-one (BA) are the most important and representative C19- and C22-steroidal materials. The optimalization of sterol production with mycobacterial phytosterol conversion has been investigated for decades. One of the major challenges is that current industrial mycobacterial strains accumulate unignorable impurities analogous to desired sterol intermediates, significantly hampering product extractions and refinements. Previously, we identified *Mycobacterium* neoaurum HGMS2 as an efficient 4-AD-producing strain (Wang et al. in Microb Cell Fact. 19:187, 2020). Recently, we have genetically modified the HGMS2 strain to remove its major impurities including ADD and 9OH-AD (Li et al. in Microb Cell Fact. 20:158, 2021). Unexpectedly, the modified mutants started to significantly accumulate BA compared with the HGMS2 strain. In this work, while we attempted to block BA occurrence during 4-AD accumulation in HGMS2 mutants, we identified a few loop pathways that regulated metabolic flux switching between 4-AD and BA accumulations and found that both the 4-AD and BA pathways shared a 9,10-secosteroidial route. One of the key enzymes in the loop pathways was Hsd4A1, which played an important role in determining 4-AD accumulation. The inactivation of the *hsd4A1* gene significantly blocked the 4-AD metabolic pathway so that the phytosterol degradation pathway flowed to the BA metabolic pathway, suggesting that the BA metabolic pathway is a complementary pathway to the 4-AD pathway. Thus, knocking out the *hsd4A1* gene essentially made the HGMS2 mutant (HGMS2^*Δhsd4A1*^) start to efficiently accumulate BA. After further knocking out the endogenous *kstd* and *ksh* genes, an HGMS2^*Δhsd4A1*^ mutant, HGMS2^*Δhsd4A1/Δkstd1*^, enhanced the phytosterol conversion rate to BA in 1.2-fold compared with the HGMS2^*Δhsd4A1*^ mutant in pilot-scale fermentation. The final BA yield increased to 38.3 g/L starting with 80 g/L of phytosterols. Furthermore, we knocked in exogenous active *kstd* or *ksh* genes to HGMS2^*Δhsd4A1/Δkstd1*^ to construct DBA- and 9OH-BA-producing strains. The resultant DBA- and 9OH-BA-producing strains, HGMS2^*Δhsd4A1/kstd2*^ and HGMS2^*Δkstd1/Δhsd4A1/kshA1B1*^, efficiently converted phytosterols to DBA- and 9OH-BA with the rates of 42.5% and 40.3%, respectively, and their final yields reached 34.2 and 37.3 g/L, respectively, starting with 80 g/L phytosterols. Overall, our study not only provides efficient strains for the industrial production of BA, DBA and 9OH-BA but also provides insights into the metabolic engineering of the HGMS2 strain to produce other important steroidal compounds.

**Supplementary Information:**

The online version contains supplementary material available at 10.1186/s12934-022-02008-8.

## Introduction

Steroids are active pharmaceutical ingredients (APIs) that are largely demanded in the clinic [1]. The most attractive and efficient strategy for producing sterol APIs is to utilize mycobacteria to transform phytosterols into androstane steroids as precursors that are used to synthesize various advanced steroid medicines through chemical and/or enzymatic modification [2–7]. Mycobacterial aerobic side chain degradation of phytosterols is fundamental to the production of androstane steroids as many mycobacteria can survive with sterols as the sole carbon source [3, 8–10]. Their sterol degradation pathways have been extensively explored and share a core pathway via 9,10-secosteroid intermediates that participate in the breakdown of steroid ring structures [11, 12].

**Scheme 1:**
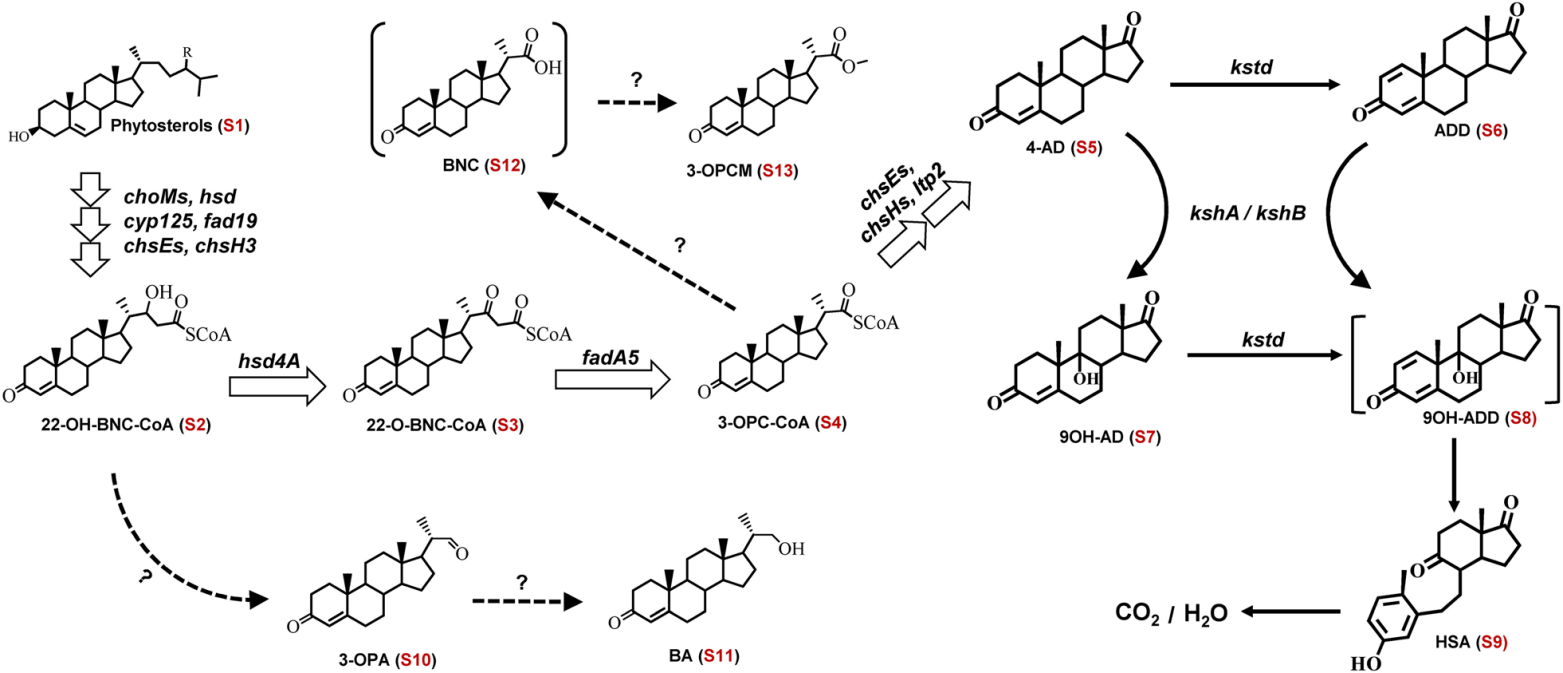
An overview of the phytosterol degradation pathway in *Mycobacterium* HGMS2. Each steroid intermediate is numbered. Open arrows refer to a known pathway for the accumulation of 4-AD (S5) and solid lines refer to the ring-broken reactions. Dashed lines refer to unknown pathways to accumulate BA (S11) and 3-OPCM (S13). The information on all steroid intermediates is summarized in Table 2

Currently, only a few sterol APIs can be produced on an industrial scale, including 4-androstene-3,17-dione (4-AD), 1,4-androstadiene-3,17-dione (ADD) and 9α-hydroxy-4-androstenediol (9OH-AD) [2, 3], requiring efficient mycobacterial cell factories to produce new sterol API on an industrial scale. In addition to traditional mutation approaches, including ultraviolet light and chemical-mediated mutation, gene and metabolic engineering have been focused on manipulating mycobacterial sterol degradation pathways to improve the phytosterol biotransformation and construct new strains to accumulate desired sterol intermediates [13–21]. The degradation pathway and the accumulation of 4-AD in mycobacteria have been thoroughly explored (Scheme 1) and many key intermediates have been identified (Table 1). Knockout of the *kstd*, *kshA* and *kshB* genes from mycobacteria enhanced 4-AD, ADD and 9OH-AD accumulation [10, 22–34]. Depletion of the *cho*, *hsd* and *kstd* genes from *M.* *smegmatis* promoted 9OH-AD accumulation [35, 36]. Deletion of the *kstd* gene from the *M.* *neoaurum* NwIB-01 strain enhanced 4-AD and ADD production [37]. Knockout of multiple *kstd* genes from *M.* *neoaurum* ATCC25795 generated an efficient 9OH-AD-producing strain [26, 32].

**Table 1:** List of steroid compounds

Compounds	Steroid name	Abbreviation
S1	Phytosterols	PS
S2	22-Hydroxy-3-oxo-cholest-4-ene-24-carboxyl-CoA	22-OH-BNC-CoA
S3	3,22-Dioxo-cholest-4-ene-24-carboxyl-CoA	22-O-BNC-CoA
S4	3-oxo-23,24-bisnorchol-4-ene-22-carboxyl-CoA	3-OPC-CoA
S5	4-androstene-3,17-dione	4-AD
S6	1,4-androstene-3,17-dione	ADD
S7	9α-hydroxy-4-androstenediol	9OH-AD
S8	9α-hydroxy-1,4-androstenediol	9OH-ADD
S9	3-hydroxy-9,10-secoandrost-1,3,5(10)-triene-9,17-dione	HSA
S10	(20S)-3-oxopregn-4-ene-20-carboxaldehyde	3-OPA
S11	22-hydroxy-23,24-bisnorchol-4-ene-3-one	BA (or 4-HBC)
S12	3-oxo-cholest-4-ene-24-carboxylic acid	BNC
S13	methyl 3-oxo-23,24-bisnorchol-4-en-22-oate	3-OPCM
S14	9,22-dihydroxy-23,24-bisnorchol-1,4-diene-3-one	9OH-DBA
S15	9,22-dihydroxy-23,24-bisnorchol-9,10-secoandrost-1,3,5(10)-triene	22-OH-HSA

22-Hydroxy-23,24-bisnorchol-4-ene-3-one (BA or 4-HP or 4-HBC) is an important precursor for the synthesis of progesterone, adrenocortical hormones [3, 13], artificial ursodeoxycholic acid and vitamin D3 [38, 39]. To date, only a few strains have been explored for the potential industrial production of BA. The deletion of the *hsd4A* gene in the *kshA-*null strain *of* *M.* *neoaurum* ATCC 25795 resulted in BA accumulation [14]. Increasing cell permeability by deleting the *MmpL3* gene, encoding a transmembrane transporter of trehalose monomycolate, was effective in enhancing BA productivity [40]. The productivity of these strains could be further improved by the use of mycobacterial resting cells [41–44]. Very recently, an engineered strain of *M.* *neoaurum* DSM 44074 could efficiently convert phytosterols to 9OH-BA [45]. These investigations have revealed that the dual competing pathways between the overwhelming C19 steroid pathway and the C22 steroid pathway are involved in phytosterol sidechain degradation [22, 24, 45–48]. Some key specific genes, such as *hsd4A* [14], *fadA5* [13, 14], *Sal* [49], *hsaG* [50] and *OpccR* [13], were involved in controlling metabolic flux between the two pathways (Scheme 1). However, the detailed mechanism is still elusive.

Previously, we characterized a 4-AD producing strain, *M.* *neoaurum* HGMS2, using genomic and enzymatic analyses and found that it had a relatively simple pathway for phytosterol degradation [51]. After knocking out its active *kstd211* and *kshA395* and *kshB122* genes, the HGMS2^*Δkstd1/*ΔkshA395^ and HGMS2^*Δkstd1/ΔkshB122*^ mutants significantly reduced the occurrence of the ADD and 9OH-AD impurities and increased the yield of the phytosterol to 4-AD conversion [52]. However, the mutants started to accumulate a small portion of the BA impurity. Thus, in this work, we dissected the two competing pathways in the HGMS2 strain, aiming to construct efficient sterol-producing strains. We bioinformatically identified two *hsd4A* genes (*hsd4A1* and *hsd4A2*) and one *opccR667* gene in the HGMS2 strain that were mainly responsible for tuning the C19-degradation pathway and the C22-degradatin pathway. Through gene knockout, we found that the roles of the *hsd4A1*, *hsd4A2* and *opccR667* genes in the HGMS2 strain and its *kstd*- and/or *ksh*-null mutants were significantly different in controlling BA accumulation. Moreover, blocking depletion of the BA pathway significantly reduced 4-AD productivity, suggesting that the BA pathway likely contributed to cell growth. Thus, our work provides useful guidance for engineering *M.* *neoaurum* HGMS2 to efficiently produce pharmaceutical sterols with tremendous potential for industrial applications.

## Materials and methods

### Strains and reagents

*Mycobacterium* *neoaurum* HGMS2 was maintained in our laboratory, and its genome sequence is available in GenBank (CP031414.1) [51]. The homology recombination vector p2NIL-Sac was constructed previously [52]. The plasmid pMV 261 was purchased from AddGene (Watertown, MA, USA). Restriction enzymes, dNTPs, and *Taq* and *Pfu* DNA polymerases were purchased from Takara Co. (Dalian, China). Other molecular biology reagents were of the highest grade and were obtained from New England Biotech (MA, USA). Genomic DNA extraction kits, plasmid purification kits and PCR purification kits were obtained from Tiangen (Beijing, China).

Standard samples of 4-androstene-3,17-dione (4-AD), 22-hydroxy-23,24-bisnorchol-1,4-diene-3-one (DBA), 9,22-dihydroxy-23,24-bisnorchol-4-ene-3-one (9OH-BA), and 22-hydroxy-23,24-bisnorchol-4-ene-3-one (BA) were prepared by Amersino (Wuhan, China). Phytosterol (98%, 410.40 Da) [52] were obtained from Hubei Goto Pharmaceutical Co. (Xiangyang, China).

### Bioinformatic prediction of phytosterol metabolic pathways

The phytosterol pathways in *Mycobacterium* HGMS2 were predicted with the KEGG pathway platform [53], integrating with previous exploration in other cholesterol-degrading strains [14, 40, 45], to query bacterial metabolic pathways, enzymes, and genes coding enzymes for steroid intermediates. A preliminary framework of the phytosterol metabolic pathways was constructed based on the genomic annotation of the *Mycobacterium* HGMS2 strain [51]. The framework includes all possible and putative enzymes, transporters and regulatory proteins that are involved in phytosterol metabolism*.*

### Preparation of recombinant and complementary mutants

Recombinant HGMS2 mutants were prepared through gene knockout and knockin strategy [52]. The upstream sequence and the downstream sequence of each target gene approximately 1 kbp in length, including *hsd4A1*, *hsd4A2*, *fadA5* and *OpccR667*, were amplified from the HGMS2 genome (Additional file 1: Tables S1 and S2) using two pairs of primers (Additional file 1: Table S3). The amplified upstream sequence and downstream sequence for each target gene were digested with two pairs of restriction enzymes, *Bam*HI/*Xba*I (or *Not*I) and *Xba*I (or *Not*I)/*Hin*dIII, respectively. Digested fragments were ligated in one step into the p2NIL-Sac vector predigested with *Bam*HI/*Hin*dIII. The ligated plasmids were amplified using *E.* *coli* DH5a competent cells and confirmed by DNA sequencing.

The recombination plasmid was electroporated into competent mycobacterial cells with a Scientz-2C electroporation System from ScienTZ (Ningbo, China) according to the procedure reported previously by Li et al. [52]. Positive recombinant colonies were transferred to 5 mL of LBT medium and cultured at 30 °C and 200 rpm for 2 days. Their genomic DNA was extracted for PCR verification. The verified mutant strains were stored at − 80 °C.

Transient expression of Kstd2 and KshA/B in the HGMS2 mutants were carried out with a modified pMV261 vector. The *kstd2* gene from *M.* *neoaurum* DSM 1381 was amplified by PCR using the primers KstD2-F and KstD2-R (Additional file 1: Table S4), and then ligated into the plasmid pMV261 at the *Eco*RI site. The *kshA/B* gene was amplified from the HGMS2 genome using KshA395-F/R and KshB122-F/R primers (Additional file 1: Table S4), and then ligated into the plasmid pMV261 at the *NdeI*-*HindIII* site. The plasmids pMV261-KstD2 and pMV261-KshA/B were electroporated into the HGMS2^*△hsd4A1*^ and HGMS2^*△kstd1/△hsd4A1*^ mutants, resulting in HGMS2^*△hsd4A1/kstd2*^ and HGMS2^*△kstd1/△hsd4A1/kshA/B*^, respectively.

The complementation of each deleted gene by the homologous expression of the *kshA226*, *OpccR667*, *hsd4A* or *fadA5* gene with pMV261 vector in corresponding mutant.

### Phytosterol transformation in small-scale fermentation

*Mycobacterium* strains were initially cultured in 5 mL of LB medium containing 0.05% Tween-80 at 30 °C for two days until the OD_600nm_ value reached 13–15. The culture was inoculated into 100 mL of fermentation medium that was composed of yeast extract (15 g/L), glucose (1 g/L), NaNO_3_ (5.4 g/L), (NH_4_)_2_HPO_4_ (0.6 g/L), β-cyclodextrin (3 g/L), TW-80 (0.05%, w/v), and phytosterol (10 g/L) and shaken at 30 °C and 200 rpm for 7 days unless otherwise mentioned. Then, 1 mL of culture broth was collected each day to extract metabolites for monitoring the process of phytosterol degradation. For comparison, only HPLC profiles on samples from the fermentation broth on the 7th day were presented when the substrate was completely consumed.

### TLC and HPLC analyses of fermentation metabolites

The fermentation broth was thoroughly mixed with ethyl acetate at a ratio of 1:1. The mixture was centrifuged at 8000×*g* for 5 min and the supernatant was collected. An aliquot of the supernatant was directly used for the thin layer chromatographic (TLC) assay. The supernatant was collected and dried by heating using a hair drier. The dried sample was dissolved in 40% methanol solution for TLC and HPLC assays as described previously [52]. The identities of HPLC peaks were confirmed by comparison with the standards of 4-AD, BA, DBA, and 9OH-BA. Each peak area was integrated with software provided by Waters and used to evaluate the compound concentration.

The mass conversion rate (*Conv*) of phytosterol to BA, DBA and 9OH-BA was estimated using the following equation:

$$Conv=\frac{Mst}{Mp}\mathrm{ \%},$$

where *M*_*st*_ and *Mp* are the weights of steroid and phytosterol, respectively.

### Phytosterol transformation in pilot-scale culture

Pilot-scale culture was conducted in a 5 L steel fermenter containing 3 L of fermentation medium. The fermentation medium was composed of yeast extract (15 g/L), glucose (8 g/L), NaNO_3_ (5.4 g/L), (NH_4_)_2_HPO_4_ (0.6 g/L), phytosterol (80 g/L), Tween-80 (0.5%, w/v), antifoam (0.3%, w/v) and lectin (3 g/L). Phytosterol was premixed with glycerol and β-cyclodextrin, and thoroughly emulsified before being transferred to the fermenter. Other materials were added to the fermenter, and water was added until the total volume of the medium reached 3 L. The medium was autoclaved in situ at 121 ℃ for 30 min, followed by cooling down to 30 ℃ with stirring at 500 rpm. The fermentation medium was inoculated with 1% (v/v) of the seed which OD_600nm_ was about 13–15. The pilot-scale fermentation was conducted at 30 ℃ with stirring at 500 rpm. The dissolved oxygen concentration and the pH value of the fermentation medium were constantly monitored in-line and automatically adjusted to maintain 50–60% and 7.5, respectively. Samples were collected every 6 h to determine the concentration of 4-AD, BA, DBA, and 9OH-BA.

## Results

### Predicted loop pathways caused residual BA in the 4-AD producing strain

**Fig. 1:**
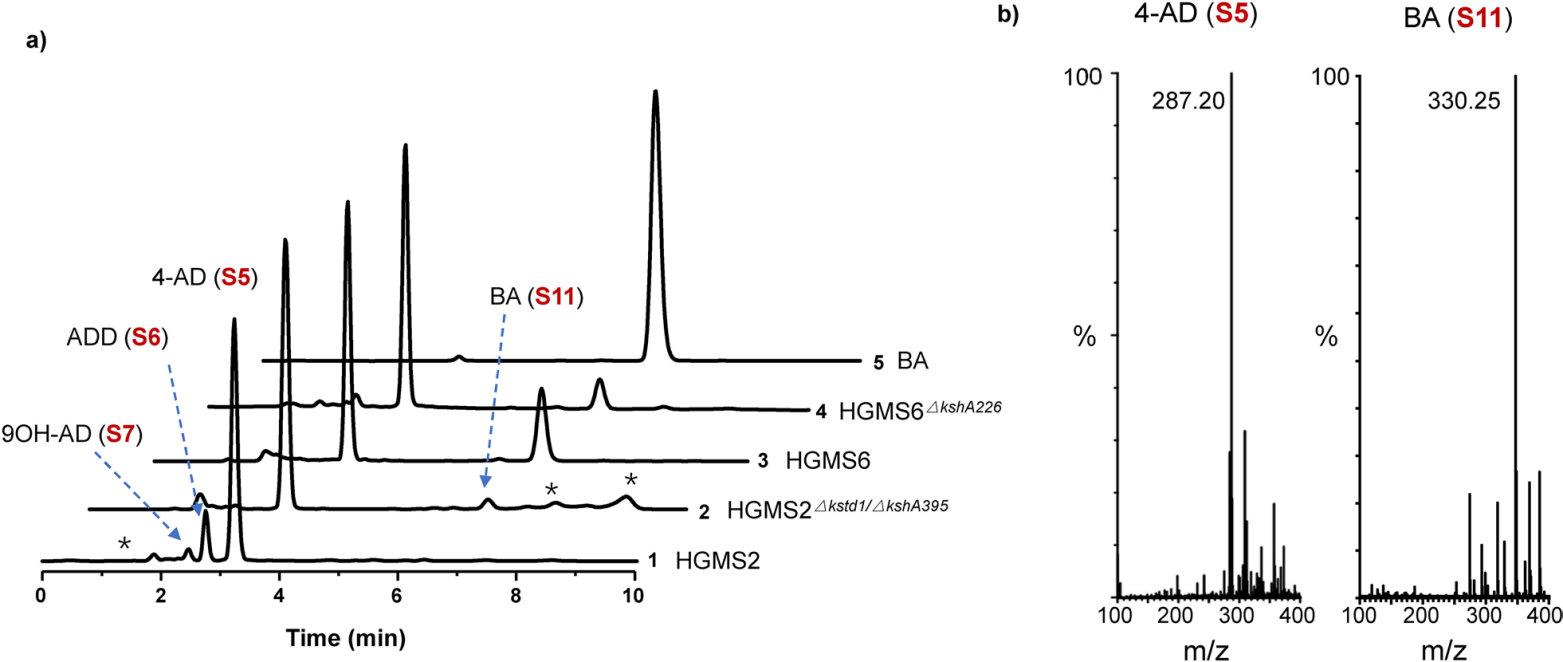
Depleting 3-ketosteroid-9α-hydroxylases caused the accumulation of BA (S11) in the HGMS2 strain. **a** HPLC profiling of the extracts from the fermentation broths of different HGMS2 mutants in comparison with that of standard BA (S15). 1: HMGS2; 2: HMGS2^*Δkstd1/ΔkshA395*^; 3: HMGS6; 4: HMGS6^*ΔkshA226*^ and 5: BA (S15). **b** Mass spectra of the 4-AD (S5) and BA (S11) peaks collected from HPLC analysis

*Mycobacterium* HGMS2 is an efficient industrial 4-AD producing strain, but its major shortcoming is that the HGMS2 strain generated certain percentage of ADD and 9OH-AD. As shown in Fig. 1a (*curve* *1*), these impurities appeared in front of 4-AD (S5) in the HPLC profile. After the deletion of active *kstd211* and *kshA395* genes, the resultant mutant, HGMS2^*Δkstd1/ΔkshA395*^, almost abolished the occurrence of ADD (S6) while significantly reducing the content of 9OH-AD (S7). However, the contents of BA (S11) and other impurities appeared to accumulate (Fig. 1a, *curve* *2*). To completely deplete the formation of any 9α-hydroxyl sterol intermediates, the *kshB122* gene, which encodes a reductase complementary to KshAs, was further knocked out from the HGMS2^*Δkstd1/ΔkshA395*^ strain, and the resultant triple mutant, named HGMS6 or HGMS2^*Δkstd1/Δksh395/ΔkshB122*^ (Table 2), indeed reduced many impurities (Fig. 1a, *curve* *3*). Contrary to our expectation, this triple mutant significantly accumulated BA (S11), although 4-AD (S5) remained the major component, which were confirmed by mass spectrometry (Fig. 1b). The percentages of 4-AD (S5) and BA (S11) were 63.5% and 23.9%, respectively. Although the content of ADD (S6) in the fermentation extract was undetectable, the trace amount of 9OH-AD (S7) was still generated (Fig. 1a, *curve* *3*). Thus, we further knocked out the *kshA226* genes encoding a 3-ketosteroid-9α-hydroxylase, which was less active for 4-AD (S5) [51]. However, as examined by HPLC profiling (Fig. 1a, *curve* *4*), this tetraplex mutant, named HGMS6^*ΔkshA226*^ (Table 2), increased the contents of 9OH-AD (S7) while the BA (S11) content was significantly reduced. These observations suggested that 4-AD (S5) and BA (S11) accumulated in parallel pathways in the HGMS2 strain, and that the two pathways shared the KstD211 and KshA/KshB-mediated ring-opening mechanism, consistent with previous investigations on other mycobacteria [14, 40, 45].

**Table 2:** Strains used in this study

Strains	Description	Main product	Impurity	Refs.
HGMS2	Wild-type strain	4-AD (S5)	ADD (S6), 9OH-AD (S7)	[51]
HGMS2^*Δkstd1*^	Deletion of *kstd211* from HGMS2	4-AD (S5)	9OH-AD (S7)	[52]
HGMS2^*Δkstd1/ΔkshA395*^	Deletion of *kshA395* from HGMS2^*Δkstd1*^	4-AD (S5)	BA(S11)	[52]
HGMS6	Deletion of the *kstd1*, *kshA395* and *kshB122* genes from HGMS2, i.e., HGMS2^*Δkstd1/Δksh395/ΔkshB122*^	4-AD (S5)	BA(S11)	[52]
HGMS6^*ΔkshA226*^	Deletion of *kshA226* from HGMS6	4-AD (S5)	BA(S11)	This study
HGMS6^*ΔkshA226/ΔopccR667*^	Deletion of both *kshA226* and *opccR667* from HGMS6	4-AD (S5)	BA(S11), Cpd1	This study
HGMS2^*Δhsd4A1*^	Deletion of *hsd4A1* from HGMS2	BA (S11)	DBA	This study
HGMS2^*Δhsd42*^	Deletion of *hsd4A2* from HGMS2	4-AD (S5)	ADD (S6)	This study
HGMS2^*ΔfadA5*^	Deletion of *fadA5* from HGMS2	4-AD (S5)	BA (S11)	This study
HGMS2^*ΔhsaG*^	Deletion of *hsaG* from HGMS2	4-AD (S5)	ADD (S6)	This study
HGMS2^*Δkstd1/Δhsd4A1ΔhsaG*^	Deletion of *hsaG* from HGMS2^*Δkstd1/Δhsd4A1*^	BA (S11)	4-AD (S5)	This study
HGMS2^*Δkstd1/Δhsd4A1*^	Deletion of *kstd1* from HGMS2^*Δhsd4A1*^	BA (S11)	4-AD (S5)	This study
HGMS2^*Δkstd1/Δhsd4A2*^	Deletion of *kstd1* from HGMS2^*Δhsd42*^	4-AD (S5)	BA (S11)	This study
HGMS2^*Δkstd1/ΔfadA5*^	Deletion of *kstd1* from HGMS2^*ΔfadA5*^	BA(S11)	Cpd2, 4-AD(S5)	This study
HGMS6^*Δhsd4A1*^	Deletion of *hsd4A1* from HGMS6	BA(S11)	4-AD(S5)	This study
HGMS6^*Δhsd4A1/Δhsd4A2*^	Deletion of *hsd4A2* from HGMS6^*Δhsd4A1*^	BA(S11)	4-AD(S5)	This study
HGMS7	Augmentation of pMV261-*kstd2* in HGMS2^*Δhsd4A1*^	DBA	BA(S11)	This study
HGMS8	Augmentation of pMV261-*kshA/B* in HGMS2^*△kstd1/△hsd4A1*^	9OH-BA	4-AD(S5)	This study

**Fig. 2:**
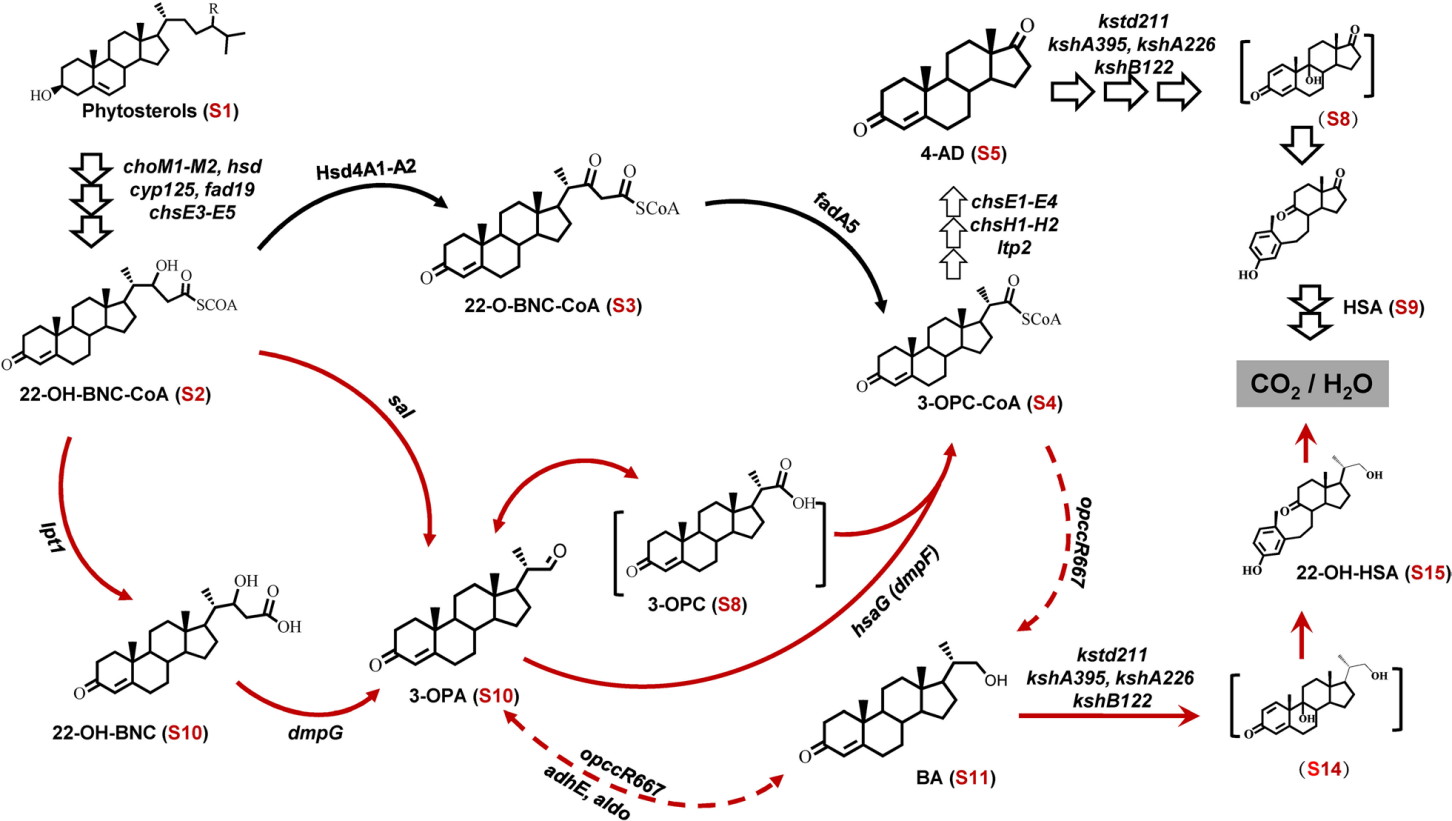
Predicted loop pathways for differentiating the 4-AD (S5) and BA (S11) accumulations during phytosterol degradation by the *Mycobacterium* HGMS2 strain. *choM*: cholesterol oxidase gene; *hsd*: 3β-hydroxyl steroid dehydrogenase/isomerase gene; *cyp125*: cytochrome P450 gene; *fad*19: acyl-CoA synthetase gene; *chs*E3-E5: long chain acyl-CoA dehydrogenase gene; *hsd*4A: β-hydroxyacyl-CoA dehydrogenase gene; *fad*A5: acyl-CoA thiolase gene; *chs*H1-H2: acyl-CoA hydratase gene; *lpt*: thioesterase gene; dmpG/Sal: aldolase genes; *hsa*G: aldehyde dehydrogenase gene; *opccR*667/*adh*E/aldo: aldehyde reductase genes; *kstd*: 3-ketosteroid-△^1^-dehydrogenase gene and *ksh*: 3-ketosteroid-9α-hydroxylase gene. The information on all steroid intermediates is summarized in Table 2

To ascertain how BA (S11) was generated in the HGMS2 strain, we predicted few possible loops in phytosterol degradation pathway, turning the phytosterol degradation pathway away from the 4-AD (S5) pathway and switching to the BA (S11) metabolic pathway (Fig. 2). Both pathways eventually converged into 9,10-secosteroid intermediates to completely degrade to CO_2_ and H_2_O. Both pathways require a series of key enzymes, including cholesterol oxidases (ChoM), 3β-hydroxyl-dehydrogenase (Hsd), 3-ketosteroid-1,2-dehydrogenase (KstD) and 3-ketosteroid-9α-hydroxylases (Ksh) and side-chain degrading enzymes, to catabolite phytosterols [22, 51, 54, 55]. As shown in Fig. 2, the phytosterol degradation pathway started to derive branch pathways from one key intermediate, i.e., 22-OH-BNC-CoA (S2). Few key enzymes were predicted to control the switching between the 4-AD (S5)_and BA (S11) pathways. The Hsd4A1 and Hsd4A2 enzymes reduced 22-OH-BNC-CoA (S2) to 22-O-BNC-CoA (S3), which was lysed to generate 4-AD (S5) by the FadA5 enzyme. 22-OH-BNC-CoA (S3) could be hydrolyzed into 3-OPA (S10) either through one step by Sal enzyme, or two steps by the Lpt1 and DmpG enzymes, via 22-OH-BNC (S10). 3-OPA (S10) is converted into BA (S11) and 3-OPC-CoA (S4) by OpccR667 and HsaG or DmpF enzymes, respectively. BA could be reversibly converted back to 3-OPA (S10) by the AdhE and Aldo enzymes, while 3-OPA (S10) could be converted into 3-OPC-CoA by the HsaG/DmpF enzyme and returns to the 4-AD pathway.

### The OpccR enzyme in the HGMS2 strain inhibited BA-accumulation

Since BA (S11) significantly accumulated during phytosterol fermentation with the HGMS6 and HGMS6^*ΔkshA226*^ mutants, we would like to block its occurrence during 4-AD (S5) accumulation. As indicated in Fig. 2, the key step before BA (S11) accumulation was the transformations of 3-OPA (S10) to BA (S11), which was catalyzed by an OpccR enzyme [13]. Through DNA blast analysis against GenBank, we identified an *OpccR* homologous gene in the HGMS2 strain, named *OpccR667* (GenBank accession No. AXK76639.1), which has 100% identity to *M.* *neoaurum* CCTCC AB2019054 (Additional file 1: Fig. S1).

**Fig. 3:**
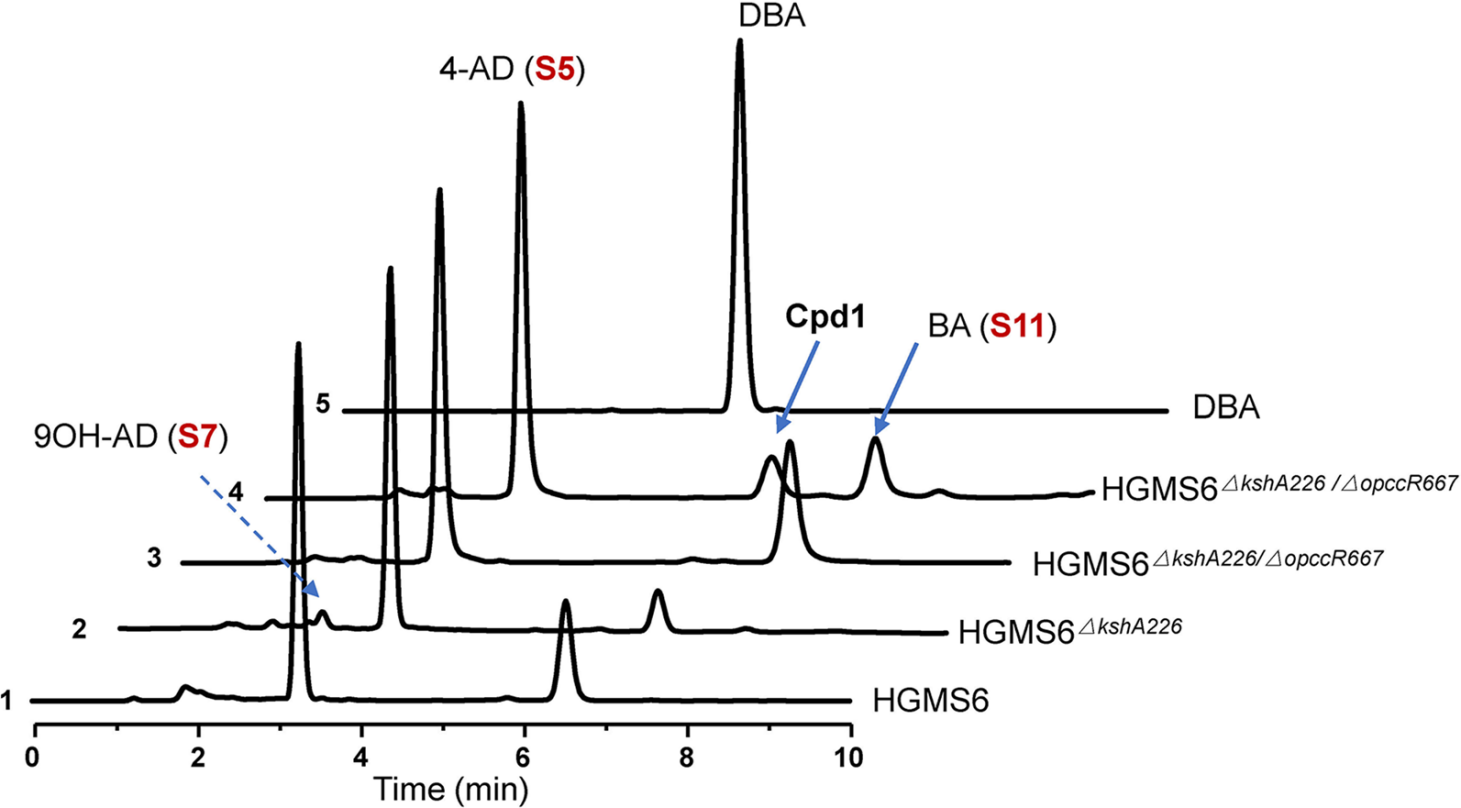
Inactivation of the OpccR667 enzyme in the HGMS strain enhanced the accumulation of BA (S11). The extracts from the fermentation broths of different HGMS6 mutants in comparison with DBA standard sample by HPLC profiling. 1: HMGS6; 2: HMGS6^*ΔkshA226*^ (6 days); 3: HMGS6^*ΔkshA226/ΔopccR667*^ (2 days); 4: HMGS6^*ΔkshA226/ΔOpccR667*^ (6 days) and 5: DBA. Cpd1 represents an unidentified intermediate

We knocked out the *OpccR667* gene from the HGMS6^*ΔkshA226*^ mutant and the resultant mutant was named HGMS6^*ΔkshA226*/*ΔopccR667*^ (Additional file 1: Table S1, Fig. S2). This mutant was evaluated for its efficiency of phytosterol conversion in small-scale fermentation. The extracts from the two- and six-day fermentation broths were evaluated by HPLC assays (Fig. 3, *curves* *3* & *4*). Unexpectedly, we found that the content of BA (S11) in the extract was not reduced, but significantly increased within two days of fermentation (Fig. 3, *curve* *3*). After six days of fermentation, its 4-AD (S5) productivity remained unchanged in the extract, while the BA (S11) content significantly decreased (Fig. 3, *curve* *4*). However, we observed that a new compound was significantly accumulated (Cpd1 in Fig. 3, *curve* *4*). Cpd1 was not DBA as it had a different retention time from that of DBA on HPLC profiling (Fig. 3, *curve* *5*). Thus, it was likely that OpccR667 reversibly catalyzed the 3-OPA/BA reaction in the HGMS2 strain. Although we could not eliminate BA (S11) from the 4-AD-producing strains, the HGMS6, HGMS6^*ΔkshA226*^ and HGMS6^*ΔkshA226*/*ΔOpccR667*^ mutants were good 4-AD-producing strains, compared with HGMS2, because BA (S11) was easily separated from 4-AD (S5) during solvent extraction. Thus, we were more interested in constructing a BA-producing strain using the HGMS2 strain to accumulate BA (S11).

### The Hsd4A1 enzyme functions as a switch for tuning the phytosterol-degrading metabolic flux between 4-AD (S5) and BA (S11) accumulation

As suggested by Fig. 2, the Hsd4A1-A2 enzymes should determine 4-AD (S5) accumulation via the transformation of 22-OH-BNC-CoA (S2) to 22-O-BNC-CoA (S3). Another enzyme should be HsaG, which transforms 3-OPA (S10) or 3-OPC (S8) to 3-OPC-CoA (S4). Deleting these three enzymes should benefit BA (S11) accumulation.

**Fig. 4:**
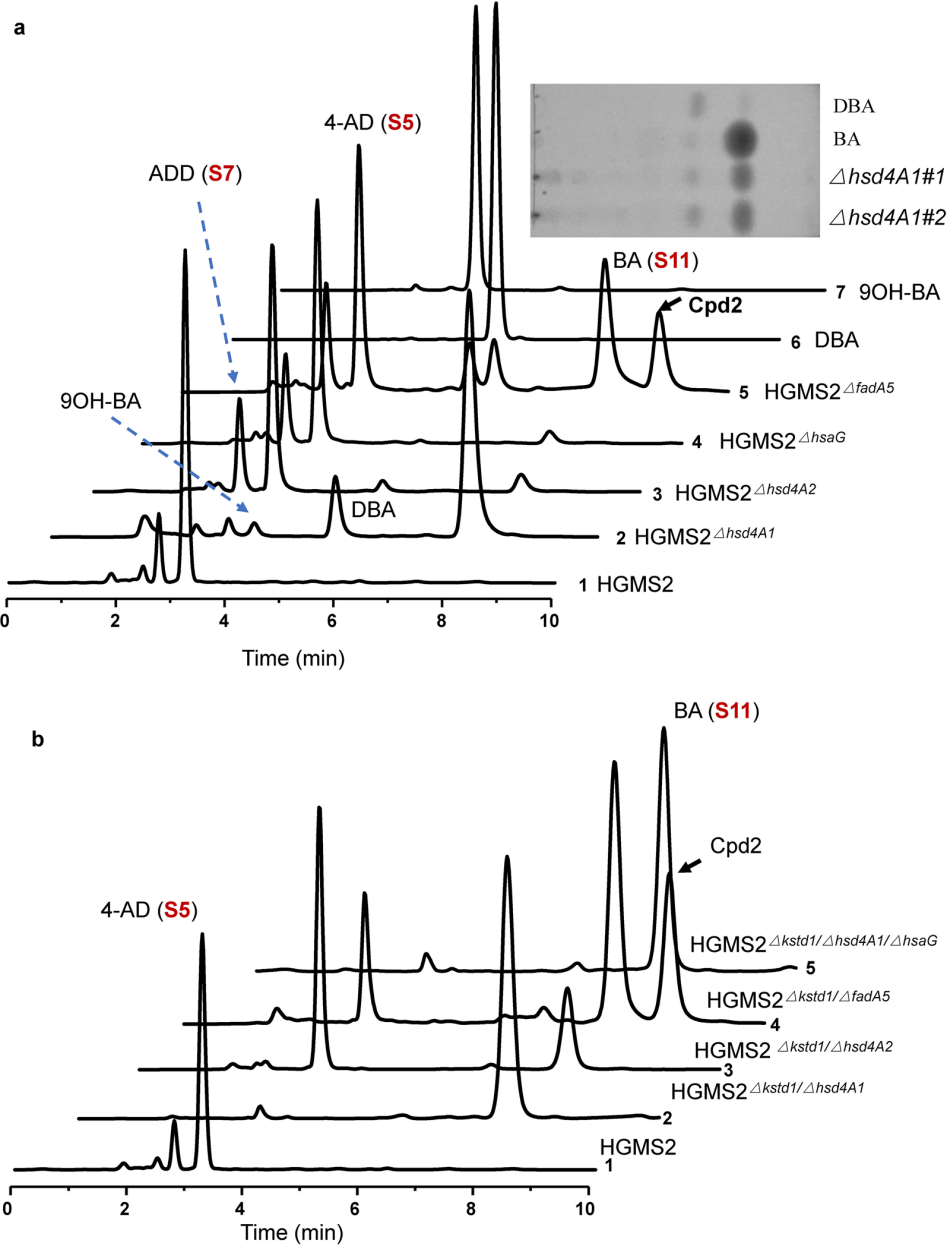
Comparison of the *hsd4A*-, *hsaG*-, *fadA5*- and *kstd*-knockout mutants for phytosterol fermentation in small-scale fermentation. **a** HPLC profiles of the extracts from phytosterol fermentation for 144 h in comparison with that of the HGMS2 strain. 1: HGMS2; 2: HGMS2^*Δhsd4A1*^; 3: HGMS2^*Δhsd4A2*^; 4: HGMS2^*ΔhsaG*^; 5: HGMS2^*ΔfadA5*^; 6: DBA and 7: 9OH-BA. Cpd2: an unidentified intermediate. Inset: TLC assay of the extracts from the fermentation broths with the HGMS2^*Δhsd4A1*^mutant. *Δhsd4A1#1* and *Δhsd4A1#2* refer to two different colonies. **b)** Effect of *kstd211*-knockout on BA (S11) accumulation. 1. HGMS2; 2. HGMS2^*Δkstd1/Δhsd4A1*^; 3. HGMS2^*Δkstd1/Δhsd4A2*^; 4. HGMS2^*Δkstd1/ΔfadA5*^ and 5. HGMS2^*Δkstd1/hsdA1/ΔhsaG*^

Thus, we started with the wild-type strain HGMS2 to explore how these enzymes affected BA (S11) accumulation. As examined by HPLC profiling the HGMS2^*Δhsd4A1*^ mutant that deleted the *hsd4A1* gene from HGMS2 (Table 2, Additional file 1: Fig. S2) almost deplete 4-AD (S5), and significantly accumulated BA (Fig. 4a, *curve* *2* and inset). Since we used the wild-type strain as the starting template, we expected to observe a few impurities such as 9OH-BA, 9OH-AD and ADD (Fig. 4a, *Curve* *2*). A mutant generated by knocking out the *hsd4*A2 gene, an *hsd4*A1 homolog named HGMS2^*Δhsd4A2*^, exhibited no significant effect on the conversion profile of phytosterol to 4-AD (S5) (Fig. 4a, *curve* *3*). The mutant generated by knocking out the *hsa*G gene, named HGMS2^*ΔhsaG*^, exhibited no significant effect on the conversion profile of phytosterol to 4-AD (S5) (Fig. 4a, *curve* *4*). These data indicated that the activity of the hsd4A2 and HsaG enzymes was much weaker than that of the hsd4A1 enzyme in HGMS2 and that hsd4A1 played an important role in switching between 4-AD (S5) and BA (S11) accumulation. Interestingly, as unexpected from Fig. 2, the mutant obtained by knocking out the *fad*A5 gene, named HGMS2^*ΔfadA5*^, promoted the accumulation of a new compound that appeared behind BA in the HPLC profile (Cpd2, Fig. 4a, *curve* *5*). Taken together, HGMS2^*Δhsd4A1*^ was a good BA-producing strain, although it still generated small amounts of impurities including DBA, 4-AD (S5), ADD (S6) and 9OH-AD (S7), as shown in Fig. 4a (*curve* *5*).

### The Hsd4A2 enzyme benefited BA (S11) accumulation in a complementary manner to the KshA395/B122 enzymes

As DBA was the major impurity during phytosterol conversion by HGMS2^*Δhsd4A1*^, HGMS2^*Δhsd4A2*^ and HGMS2^*ΔfadA5*^ strains, we would like to remove the sole 3-ketosteroid-1,2-dehydrogenase (KstD211) from these three strains. Knockout of its *kstd211* gene from the three HGMS2 mutants exhibited the expected effects on BA (S11) accumulation.

After knocking out the *kstd211* gene from the HGMS2^*Δhsd4A1*^ strain (Additional file 1: Fig. S2), HPLC profiling of its fermentation broth indicated that DBA was almost disappeared in the resultant mutant, i.e., HGMS2^*Δkstd1/Δhsd4A1*^ (Table 2), as shown in Fig. 4b (*curve* *2*), except that a small portion of 4-AD remained. Contrary to the HGMS2^*Δkstd1/Δhsd4A1*^ mutant, the HGMS2^*Δkstd1/Δhsd4A2*^ mutant in which the *kstd211* gene was knocked out from HGMS2^*Δhsd4A2*^ (Table 2) accumulated less BA (S11) (Fig. 4b, *curve* *3*). Nevertheless, DBA occurrence was significantly reduced in both HGMS2^*Δkstd1/Δhsd4A1*^ and HGMS2^*Δkstd1/Δhsd4A2*^ mutants.

When the *ktdst211* gene was knocked out from HGMS2^*ΔfadA5*^ mutant (Additional file 1: Fig. S2), the resultant double mutant, HGMS2^*Δkstd1/ΔfadA5*^ (Table 2), was also able to significantly accumulate BA, as profiled by HPLC analysis (Fig. 4b, *curve* *4*). Compared with HGMS2^*ΔfadA5*^, this double mutant showed enhanced BA (S11) accumulation and reduced 4-AD (S5) accumulation. It was notable that similar to HGMS2^*ΔfadA5*^, this double mutant continues to accumulate a high concentration of the Cpd2 compound (Fig. 4b, *curve* *4*). Furthermore, we knocked out the *hsaG* gene from the HGMS2^*Δkstd1/Δhsd4A1*^ strain. The resultant triple mutant, HGMS2^*Δkstd1/Δhsd4A1/ΔhsaG*^ (Table 1), exhibited no significant difference from HGMS2^*Δkstd1/Δhsd4A1*^, by HPLC profiling of its fermentation broth, confirming that the *hsaG* gene was inactive in the HGMS2 strain (Fig. 4b, *curve* *5*). Nevertheless, the HGMS2^*Δkstd1/Δhsd4A1*^, HGMS2^*Δkstd1/Δhsd4A2*^ and HGMS2^*Δkstd1/ΔfadA5*^ mutants could demolish the occurrence of DBA. Taken together, the Hsd4A1, Hsd4A2 and FadA5 enzymes played an important role in controlling the switching of the 4-AD pathway and the BA pathway (Fig. 2) The deletion of KstD211 not only removed DBA and ADD impurities, but also rebalanced the profiles of 4-AD and BA accumulation.

**Fig. 5:**
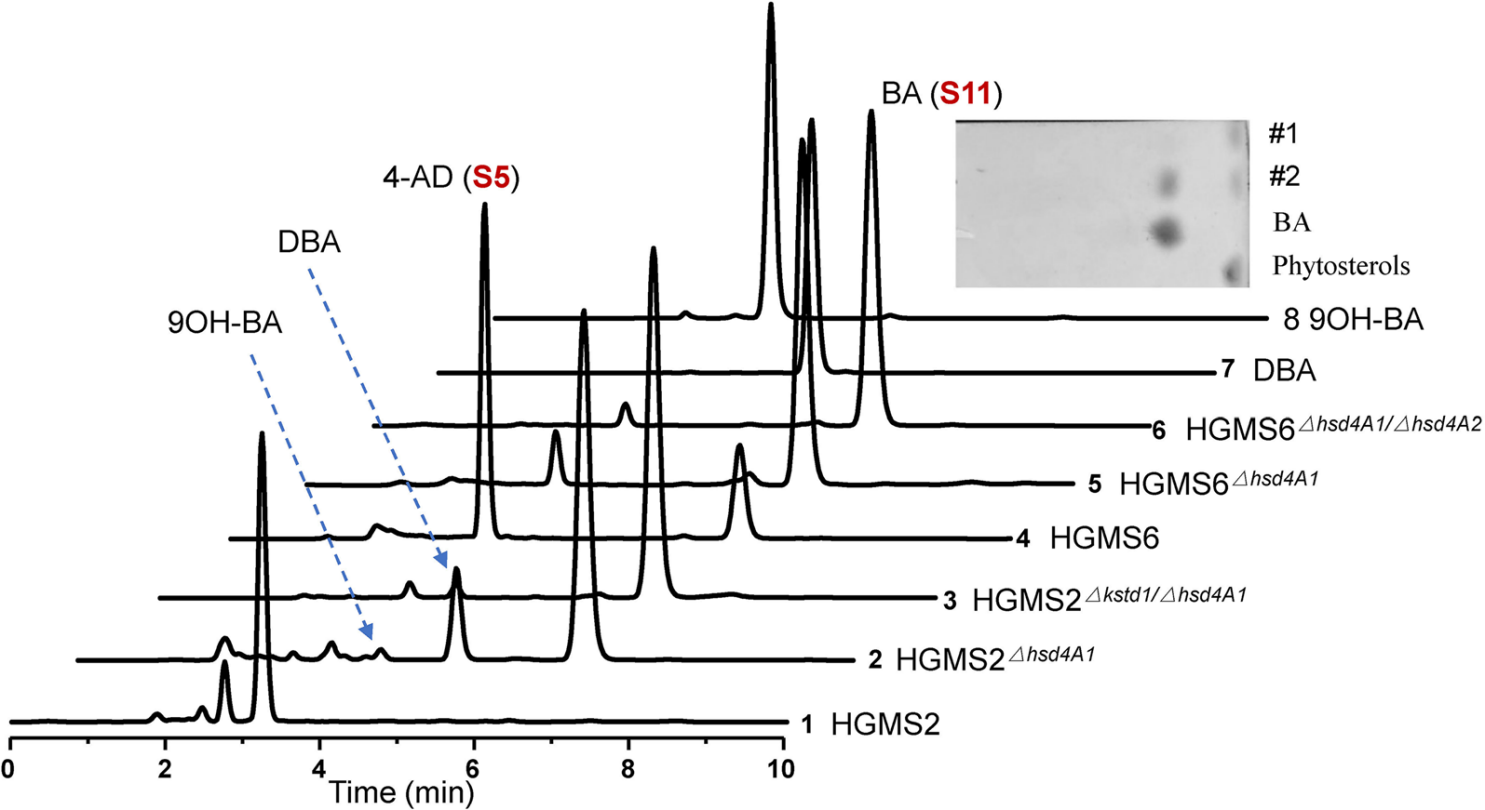
Effect of 3-ketosteroid-9α-hydroxylases on BA (S11) accumulation in HGMS2 examined by HPLC profiling. 1: HMGS2; 2: HGMS2^*Δhsd4A1*^; 3: HGMS2^*Δkstd1/Δhsd4A1*^; 4: HMGS6; 5: HGMS6^*Δhsd4A1*^ and 6: HMGS6^*ΔhsdA1/ΔhsdA2*^. Inset: TLC assay of the extracts from the fermentation broth with the HGMS6^*Δhsd4A1/ΔhsdA2*^ mutant. #1: 3 days and #2: 5 days

Thus, the HGMS2^*Δkstd1/Δhsd4A1*^ mutant could be considered to construct BA-producing strains. To maximumally reduce impurities, we further removed the 3-ketosteroid-9α-hydroxylase genes from the HGMS2^*Δkstd1/Δhsd4A1*^ mutant. Since HGMS6 or HGMS2^*Δkstd1/ΔkshA395/ΔkshB122*^ is a *kstd*- and *ksh*-null mutant (Table 2), we simply used HGMS6 as starting strain to knock out the *hshA1* gene. A resultant mutant, HGMS6^*Δhsd4A1*^, did not reduce the residual 4-AD, but increased its content as indicated by HPLC profiling (Fig. 5, *curve* 5), compared with the HPLC profile of the fermentation broth of the HGMS2^*△kstd1/△hsd4A1*^ mutant. We further knocked out the *hsd4A2* gene from the HGMS6^*Δhsd4A1*^ mutant, and found that the resultant mutant, HGMS6^*Δhsd4A1/Δhsd4A2*^ (Table 2), recovered its high productivity of BA, as indicated by HPLC profiling (Fig. 5, *curve* *6*), although a tiny portion of 4-AD remained. Thus, it was highly likely that the roles of Hsd4A1, Hsd4A2 and FadA5 enzymes in switching the 4-AD and BA pathways were dependent on the KstD and KshA/B enzymes. Thus, we selected the HGMS6^*Δhsd4A1/Δhsd4A2*^ strain as a BA-producing strain for further investigation in pilot-scale fermentation.

### Comparison of 4-AD- and BA-producing strains in pilot‑scale fermentation

Pilot-scale phytosterol fermentations were conducted in a 5 L fermenter supplied with 3 L fermentation media for HGMS6, HGMS6^*ΔkshA226*^, HGMS6^*Δhsd4A1/Δhsd4A2*^ and HGMS2^*Δkstd1/Δhsd4A1*^ (see “Materials and methods”).

**Fig. 6:**
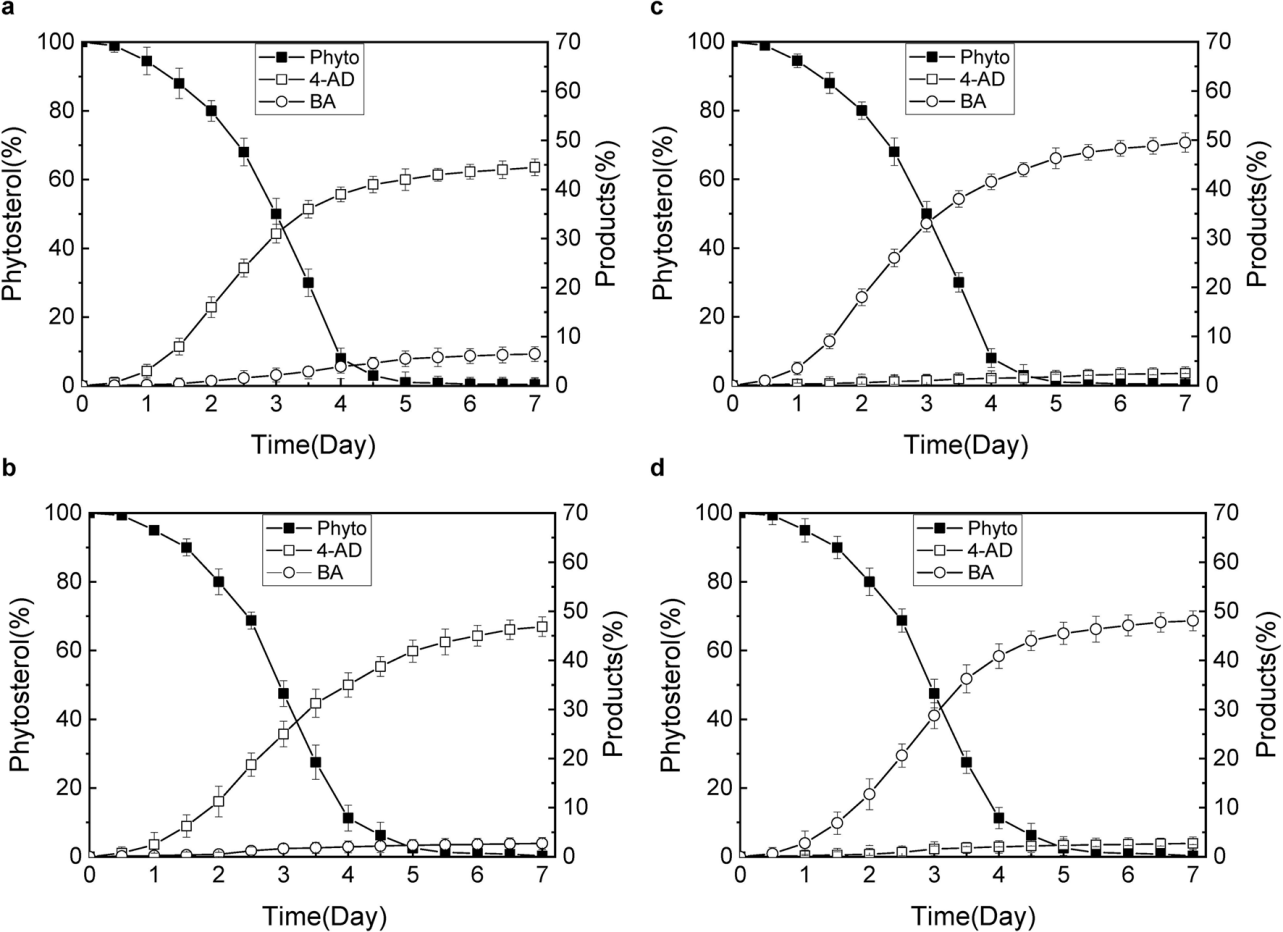
Comparison of phytosterol conversion to 4-AD (S5) and BA (S11) in pilot-scale fermentation.** a** 4-AD (S5) production by the HGMS6 strain. **b** 4-AD (S5) production by the HGMS6^*ΔkshA226*^ strain; **c** BA (S11) production by the HGMS6^*Δhsd4A1/Δhsd4A2*^ strain and **d** BA production by the HGMS2^*Δkstd1/Δhsd4A1*^ strain

As shown in Fig. 6a, the rate of phytosterol conversion to 4-AD by the HGMS6 strain increased dramatically within the first 5 days and reached 44.6% after 7 days of fermentation with 80 g/L of phytosterol. On average, the final yield of 4-AD was 37.5 ± 3.2 g/L (Fig. 6a). Accompanying the efficient production of 4-AD, the BA accumulation was also observed with yields of 6.45 g/L on average after 7 days of fermentation (Fig. 6a), resulting in the total BA contents of 8.7% on average. Compared with HGMS6, the production of BA in the metabolites of HGMS6^*ΔkshA226*^ decreased relatively, and the production of 4-AD increased slightly from 44.6% to 46.8% (Fig. 6b).

As expected, the HGMS6^*Δhsd4A1/Δhsd4A2*^ mutant exhibited an enhanced conversion rate of phytosterol to BA. As shown in Fig. 6c, the conversion rate of phytosterol to BA increased to 49.2% after 7 days of fermentation. The final yield of BA in the fermentation broth was estimated to be 39.4 ± 2.8 g/L on average (Table 3) with 80 g/L of phytosterol. Notably, 4-AD almost completely disappeared. Moreover, the HGMS6^*Δhsd4A1/Δhsd4A2*^ mutant completely catabolized the substrate with the same efficiency as the HGMS6 strain after 7 days of fermentation (Fig. 6c). Simmiar to the HGMS6^*Δhsd4A1/Δhsd4A2*^ mutant, the HGMS2^*Δkstd1/Δhsd4A1*^ mutant was also an efficient strain with high BA production (Fig. 6d, Table 3). Trace amounts of 9-hydroxyal products, such as 9OH-BA, 9OH-AD (Fig. 5, *curve* *2*), were observed due to the existence of KshA/B enzymes in the strain. Nevertheless, both the HGMS6^*Δhsd4A1/Δhsd4A2*^ and HGMS2^*Δkstd1/Δhsd4A1*^ mutants were efficient BA-producing strains.

**Table 3:** Comparison of the BA yields by a variety of *Mycobacterium* strains

Strains	Phytosterols (g/L)	Fermentation period (days)	Yield (g/L)	References
HGMS2^*△kstd1/△hsd4A1*^	10	7	8.42	This work
HGMS2^*△kstd1/△hsd4A1*^	80	7	39.26	This work
HGMS6^*△ksd4A1/△ksd4A2*^	80	7	39.46	This work
*Mycobacterium* *sp.* DSM 1381^*△kstd1*^	20	7	14.18	[56]
*Mycobacterium* *sp*. mJTU8	10	6	7.41	[13]
*Mycobacterium* *neoaurum* ATCC 25795	40	6	5.24–5.75	[14]

### Construction of DBA-producing and 9OH-BA-producing strains

Encouraged by our successful work on construct ADD- and 9OH-AD-producing strains based on the overexpression of the *kstd2* gene and *kshA395/kshB122* genes in HGMS3 and HGMS2^*Δkstd1*^, respectively [52], we would like to employ the same strategy to construct DBA-producing strain and 9OH-BA-producing strain using the HGMS2^*Δhsd4A1*^ and HGMS2^*Δkstd1/Δhsd4A1*^ mutant as templates.

**Fig. 7:**
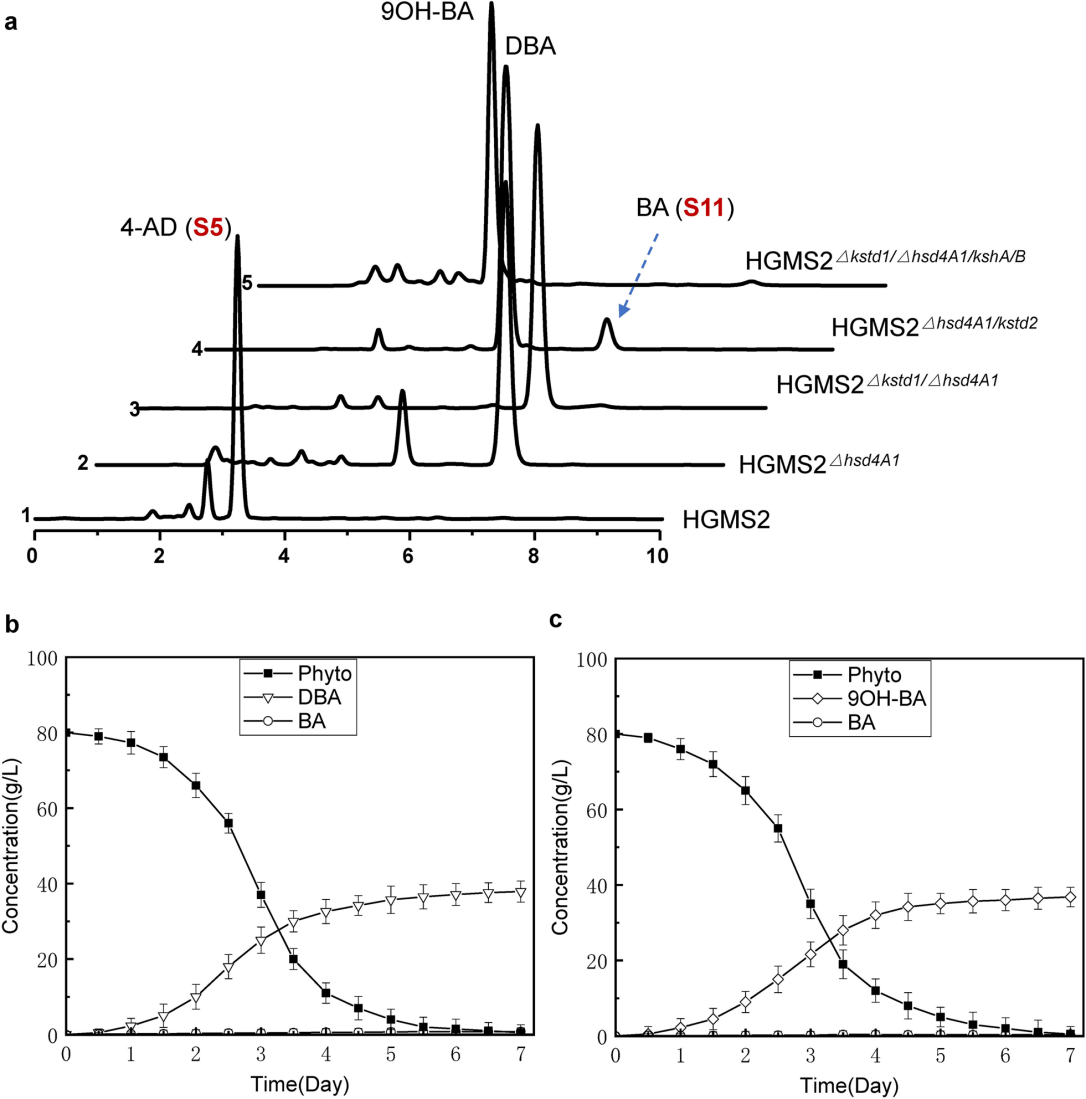
Characterization of DBA- and 9OH-BA-producing mutants for phytosterol fermentation. **a** HPLC profiles of the extracts from the phytosterol fermentation by DBA- and 9-OH-BA-producing mutants for 144 h compared with those of HGMS2^*△hsd4A1/kstd2*^ and the HGMS2^*△kstd1/△hsd4A1/kshA/B*^ mutants. 1: HGMS2; 2: HGMS2^*△hsd4A1*^; 3: HGMS2^*△kstd1/△hsdA1*^; 4: HGMS2^*△hsd4A1/kstd2*^; 5: HGMS2^*△kstd1/△hsd4A1/kshA/B*^. **b**, **c**. Time course of DBA and 9OH-BA yields by the HGMS2^*△hsd4A1/*kstd2^ and HGMS2^*△kstd1/△hsd4A1/kshA/B*^ strains

Therefore, to develop a DBA-producing strain, KstD2 was overexpressed in HGMS2^*Δhsd4A1*^ with an expression plasmid pMV261-kstD2 [52]. The resultant mutant, named HGMS2^*Δhsd4A1/kstd2*^, tested in a small-scale fermentation system using phytosterol as substrate, efficiently accumulated DBA within 7 days with a conversion rate of 68.2% and a yield of 6.17 g/L starting with 10 g/L of phytosterol (Table 2). Upon HPLC analysis (Fig. 7a), small amouint of BA and ADD were remained, no detectable 4-AD and 9OH-AD peaks were observed (Fig. 7a, *curve* 4). During pilot-scale fermentation, the conversion rate of phytosterol to DBA was 46.2% on average after 7 days of fermentation with 80 g/L of phytosterol. The final DBA yield in the fermentation broth was estimated to be 37.9 ± 3.6 g/L on average (Fig. 7b). As shown in Fig. 7b, this DBA-producing mutant completely catabolized the substrate after 5.5 days during pilot-scale fermentation.

To construct a 9OH-BA-producing strain, a previously constructed KshA1 and KshB1-expression vector, i.e., pMV261-KshA1B1 [52], was transformed into HGMS2^*Δkstd1/Δhsd4A1*^ to express cellular KshA1/KshB1, resulting in the HGMS2^*Δkstd1/Δhsd4A1/kshA1B1*^ mutant. We examined the HGMS2^*Δkstd1/Δhsd4A1/kshA1B1*^ mutant for phytosterol transformation in small-scale fermentation for 7 days, and the fermentation broth was extracted with ether acetate and evaluated by HPLC. As shown in Fig. 7a (*curve* *5*), 9OH-BA significantly accumulated in this mutant, although a small portion of BA remained. At the end of 7 days of fermentation. The final conversion rate and the yield were 67.3% and of 6.3 g/L, respectively, when starting from 10 g/L of phytosterol. With pilot-scale fermentation the conversion rate of phytosterol to 9OH-BA was 39.8% on average after 7 days of fermentation (Fig. 7c). This 9OH-BA-producing mutant completely catabolized the phytosterol substrate (80 g/L) after 7 days, with an estimated final 9OH-BA yield in the fermentation broth of 42.8 ± 3.1 g/L on average. During phytosterol transformation, we found that no BA significantly accumulated as an intermediate.

## Discussion

In this work, we dissected loop pathways that tuned the 4-AD (S5) and BA (S11) accumulations in an industrial 4-AD producing strain, *Mycobacterium* *neoaurum* HGMS2 [51]. These loop pathways (Fig. 2) were predicted based on the genomic DNA sequencing data of the HGMS2 strain in combination with previous metabolic engineering investigations of key specific genes including *hsd4A* [14], *opccR* [13], *fadA5* [14], and *hsaG* [50]. Our mutation assays on HGMS2 confirmed that different *Mycobacteria* exhibited distinct branched pathways in phytosterol-degradation, as outlined in Fig. 2. Furthermore, our gene complementation experiments confirmed that the knockout of these genes did not cause any polar effects on the phytosterol degradation pathways in the HGMS2 strain (Additional file 1: Table S5, Fig S3), indicating that these key genes play important roles in the BA (S11) accumulation.

Previously, Wei and coworkers found that inactivation of the hsd4A gene in the *kshA*-null strain of *M.* *neoaurum* ATCC 25795 enabled BA (S11) accumulation [14]. In the HGMS2 strain, we identified two homologous *hsd4A* genes, i.e., *hsd4A1* and *hsd4A2*. Similar to the sole Hsd4A enzyme in the ATCC 25795 strain, the Hsd4A1 enzyme in the HGMS2 strain determined phytosterol-degrading metabolic flux to 4-AD (S5) accumulation (Fig. 4a). Although the Hsd4A2 enzyme was much less active than the Hsd4A1 enzyme, the Hsd4A2 enzyme could also benefit BA (S11) accumulation complementary to the KshA395/B122 enzymes (Fig. 4b). Recently, Qu and coworkers found that the OpccR enzyme in *M.* *neoaurum* CCTCC AB2019054 functioned as a dual-role reductase for phytosterol catabolism, enabling efficient BA (S11) accumulation. Although the OpccR enzyme from the HGMS2 strain was identical to that of the CCTCC *AB2019054* strain (Additional file 1: Fig. S1), we found that the inactivation of the *opccR* gene in the HGMS6 strain increased BA (S11) accumulation within 2 days of fermentation (Fig. 3). It is likely that BA (S11) pathways in these mycobacterial strains are different. After 6 days of fermentation, the HGMS6 strain started to accumulate a novel intermediate (Cpd1 in Fig. 3). It was likely that the phytosterol metabolic pathway in the HGMS2 strain was more complicated than that in the CCTCC AB2019054 strain and other isoenzymes should still exist in the HGMS2 strain, needing further investigation to verify their function. Furthermore, knockout of the *fadA5* gene from the HGMS2 strain promoted the accumulation of another new intermediate (Cpd2, in Fig. 4), accompanied by an increase in BA (S11) content. On the other hand, it was likely that the knockout of the *hsaG* gene from the HGMS2 strain did not affect 4-AD (S5) and BA (S11) accumulation. However, the inactivation of the *hsd4A*, *fadA5* and *hsaG* genes in the *kstd*- and *ksh*-deficient HGMS2 strains significantly enhanced BA (S11) accumulation (Figs. 4, 5). Thus, multiple and complicated phytosterol degradation pathways exist in mycobacteria. In terms of two new compounds, Cpd1 and Cpd2, we are currently working on their structures by nuclear magnetic resonance (NMR) spectrometry. Cpd2 has been identified as a novel intermediate that accumulates in the phytosterol degradation pathway. The molecular formula of Cpd2 is C_23_H_34_O_2_ and its structure is shown in Additional file 1: Fig S4. However, the characterization of Cpd1 is still under investigation.

Nevertheless, in this work, we not only reduced the BA content in our previously constructed 4-AD producing mutant, i.e., HGMS2^*Δkstd1/Δksh395/ΔkshB122*^ [52] or named HGMS6 in this study (Table 2), but also have significantly transformed this 4-AD-producing strain to a few efficient BA-producing strains, such as HGMS2^*△kstd1/△hsd4A1*^ and HGMS6^*△hsd4A1/△hsd4A2*^. As examined in pilot-scale fermentation, the HGMS6^*Δhsd4A1/Δhsd4A2*^ mutant converted phytosterols to BA with a rate of 49.2% and a final yield of 39.4 ± 2.8 g/L within 7 days, ready for industrial application, comparable to other strains (Table 3). Furthermore, we have not observed transiently released intermediates during phytosterol degradation by those mutants created in this work (Additional file 1: Fig S5). Although the strains remain to accumulate trace amounts of 4-AD (S5), it should not be an issue for industrial application as 4-AD (S5) and BA (S11) are easily separated with solvent extraction. Because both 4-AD (S5) and BA (S11) are two important starting materials for the synthesis of sterol medicine, many mutants generated from this work can be useful tools to produce 4-AD (S5) and BA (S11) simultaneously. Furthermore, we transformed the BA-producing strains into DBA- and 9OH-BA-producing strains by overexpressing KstD2 and KshA/B enzymes, respectively.

## Supplementary Information

**Additional file 1: Table S1**. Homologous recombinant sequences for knocking out targeted genes from the HGMS2 mutants. **Table S2**. DNA sequence of key genes. Table S3. Primers for constructing knockout vectors. **Table S3**. Primers for constructing knockout vectors. **Table S4**. Primers used for gene overexpression.** Table S5**. Summary of gene complementation. **Fig. S1**. Amino acid alignment of OpccR667 with MnOpccR enzyme. MnOpccR: *Mycobacterium sp*. CCTCC AB2019054. **Fig. S2**. Generation of the* opccR667*-, *hsdA1-, hsdA2- *and *fadA5-*deficient mutants. **Fig. S3**. The complementation assays of the deleted *OpccR667, kshA226, hsd4A* and *fadA5* genes by homologous expression. Fig. S4. Structure characterization of Cpd2. **Fig. S5**. HPLC profiles of samples extracted from the fermentation broth during 5-day fermentation.

## Data Availability

Not applicable.

## Abbreviations

4-AD: 4-Androstene-3,17-dione
9OH-AD: 9α-Hydroxyl-4-androstene-3,17-dione
ADD: 1,4-Androstene-3,17-dione
BA: 21-Hydroxy-20-methylpregn-4-en-3-one
HPLC: High-performance liquid chromatography
HSA: 3-Hydroxy-9,10-secoandrost-1,3,5(10)-triene-9,17-dione
KstD: 3-Ketosteroid-1,2-dehydrogenase
Ksh: 3-Ketosteroid-9α-hydroxylase
TLC: Thin layer chromatography

## References

[1] Sultana N (2018). Microbial biotransformation of bioactive and clinically useful steroids and some salient features of steroids and biotransformation. Steroids.

[2] Fernández-Cabezón L, Galán B, García JL (2018). New insights on steroid biotechnology. Front Microbiol.

[3] Donova MV, Egorova OV (2012). Microbial steroid transformations: current state and prospects. Appl Microbiol Biotechnol.

[4] Shtratnikova VY, Schelkunov MI, Pekov YA, Fokina VV, Logacheva MD, Sokolov SL, Bragin EY, Ashapkin VV, Donova MV (2015). Complete genome sequence of steroid-transforming nocardioides simplex VKM Ac-2033D. Genome Announc.

[5] Marsheck WJ, Kraychy S, Muir RD (1972). Microbial degradation of sterols. Appl Microbiol.

[6] Wei W, Fan S, Wang F, Wei D (2010). A new steroid-transforming strain of *Mycobacterium*
*neoaurum* and cloning of 3-ketosteroid 9α-hydroxylase in NwIB-01. Appl Biochem Biotechnol.

[7] Wei JH, Yin X, Welander PV (2016). Sterol synthesis in diverse bacteria. Front Microbiol.

[8] Donova MV (2017). Steroid bioconversions. Methods Mol Biol.

[9] Malaviya A, Gomes J (2008). Androstenedione production by biotransformation of phytosterols. Bioresour Technol.

[10] Galán B, Uhía I, García-Fernández E, Martínez I, Bahíllo E, de la Fuente JL, Barredo JL, Fernández-Cabezón L, García JL (2017). *Mycobacterium*
*smegmatis* is a suitable cell factory for the production of steroidic synthons. Microb Biotechnol.

[11] Holert J, Cardenas E, Bergstrand LH, Zaikova E, Hahn AS, Hallam SJ, Mohn WW (2018). Metagenomes reveal global distribution of bacterial steroid catabolism in natural, engineered, and host environments. MBio.

[12] Bergstrand LH, Cardenas E, Holert J, Van Hamme JD, Mohn WW (2016). Delineation of steroid-degrading microorganisms through comparative genomic analysis. MBio.

[13] Peng H, Wang Y, Jiang K, Chen X, Zhang W, Zhang Y, Deng Z, Qu X (2021). A dual role reductase from phytosterols catabolism enables the efficient production of valuable steroid precursors. Angew Chem Int Ed Engl.

[14] Xu LQ, Liu YJ, Yao K, Liu HH, Tao XY, Wang FQ, Wei DZ (2016). Unraveling and engineering the production of 23,24-bisnorcholenic steroids in sterol metabolism. Sci Rep.

[15] Seidel L, Horhold C (1992). Selection and characterization of new microorganisms for the manufacture of 9-OH-AD from sterols. J Basic Microbiol.

[16] Rodina NV, Molchanova MA, Voishvillo NE, Andryushina VA, Stytsenko TS (2008). Conversion of phytosterols into androstenedione by *Mycobacterium*
*neoaurum*. Appl Biochem Microbiol.

[17] Su L, Shen Y, Xia M, Shang Z, Xu S, An X, Wang M (2018). Overexpression of cytochrome p450 125 in Mycobacterium: a rational strategy in the promotion of phytosterol biotransformation. J Ind Microbiol Biotechnol.

[18] Su L, Shen Y, Zhang W, Gao T, Shang Z, Wang M (2017). Cofactor engineering to regulate NAD(+)/NADH ratio with its application to phytosterols biotransformation. Microb Cell Fact.

[19] van der Geize R, Hessels GI, van Gerwen R, van der Meijden P, Dijkhuizen L (2001). Unmarked gene deletion mutagenesis of kstD, encoding 3-ketosteroid Δ1-dehydrogenase, in *Rhodococcus*
*erythropolis* SQ1 using sacB as counter-selectable marker. FEMS Microbiol Lett.

[20] van der Geize R, Hessels GI, van Gerwen R, Vrijbloed JW, van der Meijden P, Dijkhuizen L (2000). Targeted disruption of the kstD gene encoding a 3-ketosteroid delta 1-dehydrogenase isoenzyme of *Rhodococcus*
*erythropolis* strain SQ1. Appl Environ Microbiol.

[21] Petrusma M, van der Geize R, Dijkhuizen L (2014). 3-Ketosteroid 9alpha-hydroxylase enzymes: rieske non-heme monooxygenases essential for bacterial steroid degradation. Antonie Van Leeuwenhoek.

[22] Zhao A, Zhang X, Li Y, Wang Z, Lv Y, Liu J, Alam MA, Xiong W, Xu J (2021). Mycolicibacterium cell factory for the production of steroid-based drug intermediates. Biotechnol Adv.

[23] Li H, Wang X, Zhou L, Ma Y, Yuan W, Zhang X, Shi J, Xu Z (2019). Enhancing expression of 3-ketosteroid-9α-hydroxylase oxygenase, an enzyme with broad substrate range and high hydroxylation ability, in Mycobacterium sp. LY-1. Appl Biochem Biotechnol.

[24] Li X, Chen X, Wang Y, Yao P, Zhang R, Feng J, Wu Q, Zhu D, Ma Y (2018). New product identification in the sterol metabolism by an industrial strain *Mycobacterium*
*neoaurum* NRRL B-3805. Steroids.

[25] Shao M, Sha Z, Zhang X, Rao Z, Xu M, Yang T, Xu Z, Yang S (2017). Efficient androst-1,4-diene-3,17-dione production by co-expressing 3-ketosteroid-Δ1-dehydrogenase and catalase in *Bacillus*
*subtilis*. J Appl Microbiol.

[26] Liu HH, Xu LQ, Yao K, Xiong LB, Tao XY, Liu M, Wang FQ, Wei DZ. Engineered 3-ketosteroid 9alpha-hydroxylases in *Mycobacterium**neoaurum*: an efficient platform for production of steroid drugs. Appl Environ Microbiol. 2018; 84. 10.1128/AEM.02777-17 PMC6029100 29728384

[27] Yuan S-F, Alper HS (2019). Metabolic engineering of microbial cell factories for production of nutraceuticals. Microb Cell Fact.

[28] García-Fernández J, Martínez I, Fernández-Cabezón L, Felpeto-Santero C, García J-L, Galán B (2017). Bioconversion of phytosterols into androstadienedione by *Mycobacterium*
*smegmatis* CECT 8331. Methods Mol Biol (Clifton, NJ).

[29] Wang X, Feng J, Zhang D, Wu Q, Zhu D, Ma Y (2017). Characterization of new recombinant 3-ketosteroid-Δ1-dehydrogenases for the biotransformation of steroids. Appl Microbiol Biotechnol.

[30] Shao M, Zhang X, Rao Z, Xu M, Yang T, Li H, Xu Z, Yang S (2016). A mutant form of 3-ketosteroid-Delta(1)-dehydrogenase gives altered androst-1,4-diene-3, 17-dione/androst-4-ene-3,17-dione molar ratios in steroid biotransformations by Mycobacterium neoaurum ST-095. J Ind Microbiol Biotechnol.

[31] Xie R, Shen Y, Qin N, Wang Y, Su L, Wang M (2015). Genetic differences in ksdD influence on the ADD/AD ratio of *Mycobacterium*
*neoaurum*. J Ind Microbiol Biotechnol.

[32] Yao K, Xu L-Q, Wang F-Q, Wei D-Z (2014). Characterization and engineering of 3-ketosteroid-△1-dehydrogenase and 3-ketosteroid-9α-hydroxylase in Mycobacterium neoaurum ATCC 25795 to produce 9α-hydroxy-4-androstene-3,17-dione through the catabolism of sterols. Metab Eng.

[33] Wei W, Fan S-Y, Wang F-Q, Wei D-Z (2014). Accumulation of androstadiene-dione by overexpression of heterologous 3-ketosteroid Δ1-dehydrogenase in *Mycobacterium*
*neoaurum* NwIB-01. World J Microbiol Biotechnol.

[34] Wei W, Wang F-Q, Fan S-Y, Wei D-Z (2010). Inactivation and augmentation of the primary 3-ketosteroid-{delta}1-dehydrogenase in *Mycobacterium*
*neoaurum* NwIB-01: biotransformation of soybean phytosterols to 4-androstene- 3,17-dione or 1,4-androstadiene-3,17-dione. Appl Environ Microbiol.

[35] Fernández-Cabezón L, Galán B, García JL (2018). Unravelling a new catabolic pathway of C-19 steroids in *Mycobacterium*
*smegmatis*. Environ Microbiol.

[36] Seidel L, Hörhold C (1992). Selection and characterization of new microorganisms for the manufacture of 9-OH-AD from sterols. J Basic Microbiol.

[37] Wei W, Fan S, Wang F, Wei D (2010). A new steroid-transforming strain of *Mycobacterium*
*neoaurum* and cloning of 3-ketosteroid 9alpha-hydroxylase in NwIB-01. Appl Biochem Biotechnol.

[38] Wang J, Gu X-Z, He L-M, Li C-C, Qiu W-W (2020). Synthesis of ursodeoxycholic acid from plant-source (20S)-21-hydroxy-20-methylpregn-4-en-3-one. Steroids.

[39] Chou WL, Xinzi;Gu, Xiangdong;Li, Chenchen; Jiang, Dengyu;Wu, Shufeng. A method for synthesizing cholesterol starting from BA. vol. CN113943336A, C07J9/00 edition. China: Estern Chiuna Normal University; 2021.

[40] Xiong L-B, Liu H-H, Xu L-Q, Sun W-J, Wang F-Q, Wei D-Z (2017). Improving the production of 22-hydroxy-23,24-bisnorchol-4-ene-3-one from sterols in *Mycobacterium*
*neoaurum* by increasing cell permeability and modifying multiple genes. Microb Cell Fact.

[41] Hu Y, Wang D, Wang X, Wei D (2020). A recycled batch biotransformation strategy for 22-hydroxy-23,24-bisnorchol-4-ene-3-one production from high concentration of phytosterols by mycobacterial resting cells. Biotechnol Lett.

[42] Manosroi A, Saowakhon S, Manosroi J (2008). Enhancement of androstadienedione production from progesterone by biotransformation using the hydroxypropyl-β-cyclodextrin complexation technique. J Steroid Biochem Mol Biol.

[43] Heipieper HJ, Neumann G, Cornelissen S, Meinhardt F (2007). Solvent-tolerant bacteria for biotransformations in two-phase fermentation systems. Appl Microbiol Biotechnol.

[44] Su L, Xu S, Shen Y, Xia M, Ren X, Wang L, Shang Z, Wang M. The sterol carrier hydroxypropyl-beta-cyclodextrin enhances the metabolism of phytosterols by *Mycobacterium**neoaurum*. Appl Environ Microbiol. 2020; 86. 10.1128/AEM.00441-20 PMC7376554 32414803

[45] Yuan C-Y, Ma Z-G, Zhang J-X, Liu X-C, Du G-L, Sun J-S, Shi J-P, Zhang B-G (2021). Production of 9,21-dihydroxy-20-methyl-pregna-4-en-3-one from phytosterols in *Mycobacterium*
*neoaurum* by modifying multiple genes and improving the intracellular environment. Microb Cell Fact.

[46] Fernandez-Cabezon L, Galan B, Garcia JL (2017). Engineering *Mycobacterium*
*smegmatis* for testosterone production. Microb Biotechnol.

[47] Liu M, Xiong LB, Tao X, Liu QH, Wang FQ, Wei DZ (2018). Metabolic adaptation of *Mycobacterium*
*neoaurum* ATCC 25795 in the catabolism of sterols for producing important steroid intermediates. J Agric Food Chem.

[48] Bragin EY, Shtratnikova VY, Dovbnya DV, Schelkunov MI, Pekov YA, Malakho SG, Egorova OV, Ivashina TV, Sokolov SL, Ashapkin VV, Donova MV (2013). Comparative analysis of genes encoding key steroid core oxidation enzymes in fast-growing *Mycobacterium* spp. strains. J Steroid Biochem Mol Biol.

[49] Holert J, Jagmann N, Philipp B (2013). The essential function of genes for a hydratase and an aldehyde dehydrogenase for growth of Pseudomonas sp. strain Chol1 with the steroid compound cholate indicates an aldolytic reaction step for deacetylation of the side chain. J Bacteriol.

[50] Liu C, Shao M, Osire T, Xu Z, Rao Z (2021). Identification of bottlenecks in 4-androstene-3,17-dione/1,4-androstadiene-3,17-dione synthesis by *Mycobacterium*
*neoaurum* JC-12 through comparative proteomics. J Biosci Bioeng.

[51] Wang H, Song S, Peng F, Yang F, Chen T, Li X, Cheng X, He Y, Huang Y, Su Z (2020). Whole-genome and enzymatic analyses of an androstenedione-producing *Mycobacterium* strain with residual phytosterol-degrading pathways. Microb Cell Fact.

[52] Li X, Chen T, Peng F, Song S, Yu J, Sidoine DN, Cheng X, Huang Y, He Y, Su Z (2021). Efficient conversion of phytosterols into 4-androstene-3,17-dione and its C1,2-dehydrogenized and 9alpha-hydroxylated derivatives by engineered Mycobacteria. Microb Cell Fact.

[53] Kanehisa M, Furumichi M, Tanabe M, Sato Y, Morishima K (2017). KEGG: new perspectives on genomes, pathways, diseases and drugs. Nucleic Acids Res.

[54] Yao K, Wang FQ, Zhang HC, Wei DZ (2013). Identification and engineering of cholesterol oxidases involved in the initial step of sterols catabolism in *Mycobacterium*
*neoaurum*. Metab Eng.

[55] El-Naggar NE-A, Deraz SF, Soliman HM, El-Deeb NM, El-Shweihy NM. Purification, characterization and amino acid content of cholesterol oxidase produced by Streptomyces aegyptia NEAE 102. BMC Microbiol. 2017; 17. 10.1186/s12866-017-0988-4 PMC5372259 28356065

[56] Zhang R, Liu X, Wang Y, Han Y, Sun J, Shi J, Zhang B (2018). Identification, function, and application of 3-ketosteroid Delta1-dehydrogenase isozymes in Mycobacterium neoaurum DSM 1381 for the production of steroidic synthons. Microb Cell Fact.

