# Supplementary material for: Loop pathways are responsible for tuning the accumulation of C19- and C22-sterol intermediates in the mycobacterial phytosterol degradation pathway
Source: Microb Cell Fact. 2023 Jan 30;22:19. doi: 10.1186/s12934-022-02008-8 (PMC9885637; doi:10.1186/s12934-022-02008-8)
Supplement: Supplementary file 1 — Additional file 1: Table S1. Homologous recombinant sequences for knocking out targeted genes from the HGMS2 mutants. Table S2. DNA sequence of key genes. Table S3. Primers for constructing knockout vectors. Table S3. Primers for constructing knockout vectors. Table S4. Primers used for gene overexpression. Table S5. Summary of gene complementation. Fig. S1. Amino acid alignment of OpccR667 with MnOpccR enzyme. MnOpccR: Mycobacterium sp. CCTCC AB2019054. Fig. S2. Generation of the opccR667-, hsdA1-, hsdA2- and fadA5-deficient mutants. Fig. S3. The complementation assays of the deleted OpccR667, kshA226, hsd4A and fadA5 genes by homologous expression. Fig. S4. Structure characterization of Cpd2. Fig. S5. HPLC profiles of samples extracted from the fermentation broth during 5-day fermentation. [file 12934_2022_2008_MOESM1_ESM.docx]

***Additional file 1***

**Loop pathways are responsible for tuning the accumulation of C19- and C22-sterol intermediates in *mycobacterial* phytosterol degradation pathway**

Shikui Song^1^, Jianxin He^1^, Xiyao Cheng^1,2.3^, Meng Gao^1^, Yongqi Huang^1^ and Zhengding Su^1,3,*^

^1^Key Laboratory of Industrial Fermentation (Ministry of Education), Cooperative Innovation Center of Industrial Fermentation (Ministry of Education & Hubei Province) and Hubei Key laboratory of Industrial Microbiology, Hubei University of Technology, Wuhan 430068, China.

^2^School of Light Industry and Food Engineering, Guangxi University, No. 100, Daxuedong Road, Xixiangtang District, Nanning, Guangxi, 530004, China.

^3^Wuhan Amersino Biodevelop Inc., B1-Building, Biolake Park, Wuhan, Hubei, 430075, China

*Corresponding Author: Zhengding Su, Email: zhengdingsu@hbut.edu.cn, Tel.: 86-156-23901978, ORCID: 0000-0003-3558-001X; Xiyao Cheng, Email: xiyaocheng@gux.edu.cn, ORCID iD: 0000-0003-2161-1743.

**Keywords:** 1,4-androstadiene-3,17-dione (ADD); 22-hydroxy-23,24-bisnorchol-4-ene-3-one (BA); 3-ketosteroid-1,2-dehydrogenase (KstD); 3-ketosteroid-9α-hydroxylase (Ksh); 4-androstene-3,17-dione (4-AD); 9α-hydroxyl-4-androstene-3,17-dione (9OH-AD); 3-hydroxy-9,10-secoandrost-1,3,5(10)-triene-9,17-dione (HSA); biotransformation; cholesterol oxidases (Cho); monooxygenase (Mon); bioconversion; phytosterols and *Mycobacterium sp*.

**Mass spectrometric and nuclear magnetic resonance spectrometric assays**

To isolate Cpd2, the fermentation broth was extracted with 3 volumes of ethyl acetate. After the mixture was centrifuged at 12,000 ×g for 15 min, the supernatant was separated and dried under reduced pressure. The powered products were dissolved in ethyl acetate and applied to a silica gel column. The column was eluted by petroleum ether and ethyl acetate (5:1, v/v) to separate the products. Then, the elutes was purified by a semi-preparative high performance liquid chromatography (HPLC) equipped with C18 reversed phase column (4.6 ×150 mm, SunFire^TM^) with a mixture of methanol and water at a ratio of 80:20 (v/v) as the mobile phase at a flow rate of 1 mL/min. Analytes were simultaneously detected with UV at 254 nm. The Cpd2 peak was collected and dried with freeze dryer for further use.

The dried Cpd2 sample was dissolved in methanol solution and was analyzed by a Q Exactive™ UHMR Hybrid Quadrupole Orbitrap™ Mass Spectrometer (Thermo Scientific, USA). Masses were recorded over a 50-500 m/z range in positive mode.

10 mg of dried Cpd2 sample was weighed and completely dissolved in 0.6 mL CDCl_3_ and loaded into an NMR tube. The ^1^H and ^13^C NMR spectra were recorded at 298 K on a Zhongke-Niujin Quantum-I Plus 400 spectrometer (Zhongke-Niujin, Wuhan, China). Data was processed and displayed using Topspin 3.6 (Bruker, Germany). Tetramethyl silane (TMS) served as the internal served as the internal standard in CDC3 and chemical shift (δ) are shown as parts per million (ppm) relative to TMS.

**Table S1. Homologous recombinant sequences for knocking out targeted genes from the HGMS2 mutants**

| **Targeted gene** | **Mutant strain** | **GenBank Sequence ID & locus** |
| --- | --- | --- |
| *hsd4A1* | HGMS2*^Δkstd1/Δhsd4A1^* | GenBank: CP031414.1 |
|  |  | 263413-264324 |
| *hsd4A2* | HGMS2*^Δkstd1/Δhsd4A2^* | GenBank: CP031414.1 |
|  |  | 157380-158285 |
| *fadA5* | HGMS2*^Δkstd1/ΔfadA5^* | GenBank: CP031414.1 |
|  |  | 159520-160683 |
| *opccR667* | HGMS6*^ΔkshA226/ΔopccR667^* | GenBank: CP031414.1 |
|  |  | 3751616-3753619 |

**Table S2. DNA sequence of key genes**

| **Gene** | **Strain** | **DNA sequence** |
| --- | --- | --- |
| *hsd4A1* | *Mycobacterium sp.* HGMS2 | ATGAACGACAACCCGATCGACCTGTCCGGAAAGGTTGCCGTCGTGACCGGCGCGGCCGCCGGCCTCGGTCGTGCCGAGGCGATAGGCCTGGCGCGCGCCGGTGCGACGGTCGTGGTCAACGACATGGCCGGCGCGCTGGACTCCTCCGATGTGCTGGCCGAGATCGAGGCCGTCGGGTCCAAGGGTGTCGCCGTCGCCGGTGATATCAGTGCGCGCAGCACGGCCGACGAGCTCGTCGAGACCGCCGACCGGCTCGGGGGACTGGGCATCGTGGTCAACAACGCGGGCATCACCCGGGACAAGATGCTGTTCAACATGTCCGACGAGGACTGGGACGCGGTGATCGCCGTGCATCTGCGC  GGGCACTTCCTGCTGACCCGCAATGCCGCGGCGTACTGGAAGGCGAAGGCCAAGGACTCCGCCGAAGGGCGGGTCTACGGGCGCATCGTCAACACCTCTTCGGAAGCCGGGATCGCCGGACCGGTGGGCCAGGCCAATTACGGTGCGGCCAAGGCCGGTATCACGGCCTTGACCCTGTCGGCGGCGCGCGGGCTGAGCAGGTACGGGGTGCGGGCCAATGCCATCGCGCCCCGTGCCCGCACCGCCATGACCGCTGGCGTGTTCGGTGATGCACCCGAGCTGGTCGACGGACAGGTCGATGCCCTCTCACCGGAGCATGTCGTCACGCTCGTGACCTACCTGTCCTCGCCCGCGTCCGAGGATGTCAACGGGCAGCTGTTCATCGTGTACGGGCCCACGGTCACCTTGGTCGCGGCCCCGGTGGCCGCGCAGCGGTTCGATGCCGTCGGTGACGCCTGGGACCCCGCGGCGTTGAGCGCCACGCTCGGTGACTTCTTTGCTAAAAGGGATCCGAATATCGGCTTCTCCGCCACCGAGCTCATGGGCTCTTGA |
| *hsd4A2* | *Mycobacterium sp.* HGMS2 | ATGGGTTTGCTCGACGGCCGGGTAGTCATCGTGACGGGCGCAGGCGGCGGCATCGGCCGGGCGCATGCGCTGGCCTTCGCCGCGGAGGGTGCACGGGTCGTGGTCAACGACATCGGTGTCGGCCTGGACGGCTCACCGGCCGGCGGTGGTAGCGCCGCGCAGGGCGTCGTCGACGAGATCATCGCCGCCGGGGGCGAGGCGGTGACCAGCGGCGCCAATGTCGCCGACTGGGCGCAGGCCGAGGGATTGATCCAGACCGCGGTGGATTCATTCGGCGGGCTCGACGTTCTGGTCAACAACGCCGGCATCGTGCGCGACCGGATGTTCGTCAACGCCACCGAGGAGGAGTTCGACGCCGTCACGGCCGTGCATCTCAAGGGGCATTTCGCCACCATGAAACATGCCGGGGCATACTGGCGCGCCCAGTCCAAGGCCGGCAAGACGGTGGATGCCCGCATCATCAACACCTCCTCCGGCGCGGGCCTGCAGGGCAGCGTCGGGCAGGCGACCTACAGCGCGTCCAAGGCCGGTATCGCCGCGCTCACCCTGGTCGCCGCGGCGGAGATGGGTCGGATCGGTGTGACCGCCAACGCCATTGCGCCCTCGGCCCGTACCCGGATGACCGAGACGGTCTTCGCGGACATGATGGCCACCCAGGATTCGGCCTTCGACACCATGGCTCCGGAGAACATCTCGCCGCTGGTGGTGTGGCTGGGCAGC  GTCGAGTCGCGTGAGGTGACCGGGCGGGTGTTCGAGGTCGAGGGCGGGATCATCCGGGTTGCCGAGGGCTGGGCACGCGGTGCCGAGGTCGACAAGGGCGCGCGCTGGGACCCGGCCGAGCTGGGGCCCGTGGTCGGCGATCTGCTGGCCAAGTCGCGCACCCCGCTGCCGGTCTTCGGGGCCTGA |
| *fadA5* | *Mycobacterium sp.* HGMS2 | ATGGGTAATCCTGTCATCGTCGAAGCCACCCGCAGCCCCATCGGCAAGCGCAACGGCTGGCTGTCCGGGCTGCATGCCACCGAACTCTTGGGCGCCGTGCAGAAGGCGGTCGTCGAGAAGGCCGGGATCGACGCCGGCGATGTCGAACAGCTCATCGGCGGCTGCGTCACCCAGTACGGCGAGCAGTCCAACAACATCACCCGGGTCGGGTGGCTGACCGCGGGATTGCCCGAGCACGTGGGTGCCACCACCATCGACTGCCAGTGCGGCAGCGCCCAGCAGGCCAACCATCTGATCGCGGGTCTGATCGCCACCGGTGCGATCGACATCGGCATCGCGTGCGGTATCGAGGCGATGAGC  CGAGTAGGCCTCGGCGCCAATGCCGGCCCCGATCGCGGTCTCATCCGCGCATCGTCATGGGACATCGACATGCCCAATCAGTTCGAGGCCGCCGAGCGGATTGCCAAGCGCCGCGGGATCACCCGGGCCGACCTGGACGCGTTCGGGCTCGCCTCGCAGGCCAAGGCCAAGCAGGCCTGGGCCGAGGGTCGCTTCGATCGGGAGATCTCACCCATCTCGGCGCCCGTGCTCGACGAGAACAAGCGTCCGACCGACGAATGGGCGCTCGTGACCCGCGACCAGGGCCTGCGCGACACCACGGCGGAGGGTTTGGCCGCGCTGAAGCCGGTGATGGAAGGTGCCATGCACACCGCGGGAACCTCCTCACAGATCTCCGATGGCGCGGCGGCGGTGCTGTGGATGGACAGCGATGTGGCCAAGGCCCACGGCCTCACGCCGCGCGCCCGGATCGTCGCGCAGGCCAATGTCGGCGCCGAGACCTACTACCACCTCGACGGCCCGGTGCAGTCGACCGCCAAGGTGCTGGAGAAGGCCGGGATGAAGATGGGCGATATCGACCTCGTCGAGATCAACGAGGCTTTCGCCTCCGTGGTGCTGTCCTGGGCGCAGGTGCACGGGGCGGACATGGACAAGGTCAACGTCAACGGCGGCGCCATCGCGCTGGGGCACCCCGTCGGCTCCACCGGCGCCCGGTTGATCACCACCGCGCTGCACGAGTTG  GAACGCACCGGCAAGAGCACCGCGCTCATCACGATGTGCGCCGGTGGCGCGCTGAGCACGGGCACCATCATCGAAAGGATCTGA |
| *opccR667* | *Mycobacterium sp.* HGMS2 | ATGGCCCGCATGCATTATGTCGTTACCGGCGGTACCGGGTTTATCGGCAGCCGGGTCATCGCCCGCCTCCTGGCACGAGATCCCGAGGCACGGGTCTCGGTGCTCGTGCGACGCGGATCGCTGCACCGCTTCGAAAAGCTCGCCGCGGGTTGGGGTCCGCGGGTGAATGCGCTGGTAGGTGACCTCACCGCACCGGACCTGGGTCTGGACGGCGCCGAGGTCGACCCCGTCGATCATGTGGTGCACTGCGGCGCGATCTATGACATGACCGCACCCGAGGGCATCCAGCGCGCCGCCAATGTCGAGGGCACCCGCGCGGTCATCGCGGCAGCCCGCAAATGGGGAGCGACCCTGCACCACGTGTCCTCGATCGCGGTGGCCGGCGATTTCGTCGGTGAGTACACCGAGGACGATTTCGACGTCGGCCAGTATCTGCCCACCCCGTATCACCAGACCAAGTTCGAGGCCGAGGCATTGGTGCGCGCCGAACCCGGACTGCGCACGCGGATCTACCGTCCGGCCGTCGTGGTCGGCGATTCCCGCACCGGCGAGATGGACAAGGCCGACGGGCCGTACTACTTCTTTCCCATCCTGGCCAAGCTCGCTGTGCTGCCGGGGTTCACCCCGATGGTGCTGCCCGATACCGGCCGGACGAACATCGTCCCGGTCGACTTCGTGGTCGACGCCATGGTCCACCTGATGCACATCGCCGACGGCGATGGCCGCACGTTCCATCTCACGGCTCCGAAAACCATTGGGCTGCGCGGCATCTACCGTGGTGTGGCCAAGGCCGCCGGGTTGCCGCCGCTGGTCGGTTCGCTCCCCGGGGCCGCCGTATCCCCCGTCCTCAAGGCAACCGGCCGCGCCAAGATCGTGCGCAACATGGCGGCCACCCAACTCGGGGTGCCCGCCGAGATCCTCGATGTCGTCGACCTCGCACCGACGTTCGTCGCCGATCGCACCACGAACGCACTCGCGGGAACGGGTATCGAGGTGCCCGAATTCGCCTCGTACGCACCGAAGTTGTGGAGATTCTGGGCGCAGAACCTGGACCCGGACCGCGCTCGTCGCGATGATCCGAAGGGCCCGCTGGTGGGCAGGCATGTCGTGATCACCGGGGCGTCCAGCGGTATCGGACGGGCCTCGGCCGTCGCCGTGGCCCGCCGCGGCGCCACGGTCTTCGCGTTGGCCCGCAACGCCGATGCACTCGACGAACTGATCGCCGAGATCCGCGCCGAGGGAGGGGACGCCCACGCTTTCACCTGTGACGTCACCGATTCCGGCTCGGTGGAGCACACGGTCAAGGACATTCTCGGGCGGTTCGGCCACGTCGACTACCTGGTGAACAATGCCGGTCGGTCGATCCGGCGGTCGGTGTCGGCCTCGACCGACCGGCTGCACGATTACGAACGGGTGATGGCGGTCAACTATTTCGGCGCCGTGCGGATGGTGCTTGCGCTGCTGCCGCACTGGCAGGAACGGCGGTTCGGACACGTGGTCAACGTGTCCAGCGCCGGCGTGCAGGCCAACAGCCCCAAGTACAGCGCCTACCTGCCGACCAAGGCGGCCCTCGACGCGTTCTCCGAAGTGGTCGGCACCGAAACACTGTCCGATCACATCACTTTCACCAACATCCACATGCCGTTGGTGCGCACCCCGATGATCGCGCCATCGCGCCGGCTCAATCCGGTTCCACCCATCACCGCCGAGCACGCCGCGGCGATGGTGGTGCGCGGGCTGATCGAGAAGCCGTCGCGCATCGACACCCCACTGGGCACGCTGGCCGATCTGGGCACCTACTTCACGCCACGACTGTCCCGGCGCGTGCTGCATCAGCTCTATCTGGGTTACCCGGATTCCGCGGCGGCCCGCGGCCAGGCACCCGAGGAAGAGGTGGCCGTGCCAGCGCGCCAGCCCCGGCGCCCGGCGCGCGCTGTGCGCGCTGTGCGGATGCCGCGACCGGTCAAGCGCGCGGTCCGAGCGATTCCGGGTGTGCACTGGTAG |
| *kstd2* | *Mycobacterium sp.* DSM1381 | ATGACCGATCAGAACAACATCACCGTCGACCTCGTCGTCGTCGGCTCGGGTACCGGGATGGCGGCAGCATTGGCTGCCCACGAGCTGGGAATGTCGACGCTGATCGTCGAGAAGAGCGCCTATGTCGGTGGTTCGACGGCTCGCTCCGGCGGTGCCTTCTGGCTTCCCGGCAGCTCCATTCTCAAGGACGCCGGTTCGGCGGACACTCCGGCCAAGGCGCGCACCTACCTTGAAGCACTCGTCGGTGACGACGTCTCACCCGAACGCGCACGCACTTTCATCGATCAGATCCCCGCGACCATCGACATGTTGCGTCGCACCACCCCGATGAAGTTCATGTGGGCCAAGGGATATTCGGACTACCACCCGGAGAGGCCAGGAGGCAGTGCGGTGGGCCGGACCTGTGAGTGTCGCCCGTTCGACACTGCGGTCCTCGGTCCAGAGCTGGCGCGGCTACGACCTGGAGTGATGAAGTCATCGTTCCCGATGCCGGTCACCGGCGCCGATTACCGTTGGCTGAACCTGATGGCCCGCACCCCGCGCAAGTCCTGGCCGCGGATCATGCTGCGGGCCATGCAGGGTGTCGGCGGTTTGGCCCTGCGGCGCCGGTACGCCGCAGGCGGCCAGGCCTTGGCGGCCGGGATGTTCGCCGGCGTGCTGCAGGCGGGGATCCCGGTGTGGACCGATTCGACGGTGACCGAGCTCATCACCGATGGTGGGCGGGTGACCGGCGCGCGGGTGCTGCGCGAGGGATCGGCCGTGACCGTCACCGCACGCCGTGGCATCGTGCTGGCCACCGGCGGTTTCGACCACGAGATGAATTGGCGGCGGAAGTTCCAGTCCGAGCTCCTCGGTGAACATCTCAGCCTTGGGGCCGAGAGCAATACCGGCGATGGCATCCGGCTCGCCCAGGACCTGGGCGCAGGCACCGGACTGATGGACCAGGCATGGTGGTTTCCGGCCTTTGCTCCGCTGCCTGGCGGGGATCCCACCGTGATGCTGGCCGAGCGGTCGCTGCCCGGCTGCCTGCTGGTAGACCAGACCGGTGAGCGCTTCATCAACGAGGCCACCGACTACATGTCCTTCGGACAGCAGCTGCTGCGTCGCGAACACGCGGGCAATCCGGTCGAGACGATGTGGATGATCTTCGATCAGCGCTACCGGAACAGCTATCTGCTTGCCGCCGAACTATTTCCACGAATGCCGATCCCACAGAGTTGGTACGACGCCGGGATCGCGCACCGCGGCACGGATGCGGAAGCACTGGGCCGCCAGATCGGTTTCGATCCCGCGACGTTGGTCGCCACGATCGAGCGGTTCAACGGACTCGCCGATGCCGGTGTCGACGCCGACTTCCAGCGCGGCGCGAGCGCCTACGACCGCTACTACGGCGACCCGACGATCACGCCCAACCCGAACCTGCGACCGCTGGATCCCGGCCCGCTGTACGCCGTCAAGGTCGTGCTGAGCGACCTGGGCACCTGTGGTGGGGTCCTGTGCGACGTGAACGGCCGGGTTCTGCGCGAAGACGGAGTGCCCATCGACGGTCTGTACGCGATCGGCAATACCGCGGCCAACGCATTCGGCAAGACCTACCCGGGCGCGGGCGCGACCATCGCGCAGGGGCTGGTGTACGGCCATGTTGCCGCGCAGCATGCCGCCGGACACACCTGA |
| *kshA395* | *Mycobacterium sp.* HGMS2 | ATGTCCATTGATACCGCACGCTCTGGTTCGGACGACGATGTCGAGATCCGCGAAATTCAAGCTGCTGCAGCTCCTACACGCTTCGCCCGCGGGTGGCACTGCTTGGGCTTATTGCGCGACTTTCAGGATGGCAAACCCCACTCCATCGAGGCGTTTGGGACTAAACTGGTGGTTTTCGCCGATTCGAAAGGGCAGTTGAACGTTCTGGACGCCTATTGTCGTCACATGGGAGGAGACTTGAGCCGCGGCGAGGTTAAGGGTGATTCTATTGCTTGTCCCTTTCATGATTGGCGCTGGAATGGAAAAGGAAAATGTACTGACATTCCCTACGCCCGCCGTGTGCCTCCGATTGCAAAGACACGCGCTT  GGACTACTTTAGAGCGTAACGGTCAACTGTATGTATGGAATGACCCCCAAGGAAATCCTCCGCCGGAGGATGTGACTATCCCTGAAATTGCTGGGTACGGAACTGATGAGTGGACCGACTGGAGTTGGAAATCACTTCGCATCAAAGGTTCTCACTGTCGTGAGATTGTCGATAACGTAGTAGACATGGCCCATTTTTTTTATATTCACTATTCTTTCCCGCGCTACTTTAAGAACGTTTTTGAAGGCCACGTTGCTAGCCAATTCATGCGCGGTCAGGCGCGTGAAGATGTTATTTCGGGTACCAACTACGATGATCCTAATGCAGAATTACGCAGCGAGGCAACATACTTCGGCCCTAGCTACATGATCGATGACTTGGAAAGCGACGCGAATGGACAAACAATTGAGACCATCTTAATTAACTGTCACTACCCGGTGTCGAACAACGAGTTCGTTTTGCAGTACGGCGCAATCGTGAAAAAGTTGCCCGGCGTCAGTGATGAGATCGCGGCCGGGATGGCTGAGCAATTCGCGGAAGGAGTTCAGCTTGGCTTTGAACAAGACGTGGAGATTTGGAAAAATAAGGCACCGATCGATAATCCCCTGTTGAGCGAAGAGGACGGGCCTGTCTACCAATTGCGTCGTTGGTACCAACAATTTTACGTAGACGTCGAAGACATCACTGAAGATATGACAAAGCGCTTCGAGTTCGAGATCGACACTACACGCGCCGTTGCCAGCTGGCAGAAGGAGGTCGCTGAGAATCTTGCCAAGCAGGCAGAAGGCTCAACCGCTACTCCCTAAGAATTCGAGCTCGGCGCGCCTGCAGGTCGACAAGCTTGCGGCCGCATAATGCTTAAGTCGAACAGAAAGTAATCGTATTGTACACGGCCGCATAATCGAAATTAA |
| *kshB122* | *Mycobacterium sp.* HGMS2 | GTGACGGAGGAACCGCTCGGCAGCCATGTGCTGGAACTGGAGATCGCCGCCGTCGTCGAGGAGACCGCCGATGCCCGGTCGCTCGTGTTCGACATCCCGGCCGGCAGCGATATGCCCGCCGAGAGGTTGCGGTACTCGCCCGGCCAGTTCCTGACGCTGCGCGTGCCCAGCGAGCGGACCGGATCGGTGGCCCGCTGCTACTCCCTGTCGAGCTCGCCCGCACACGGCGAGAAGCTGACCGTGACCGTCAAGCGGACCGCCGACGGGTACGCGTCGAATTGGCTGTGCGACAACGCCCATCGCGGTATGCGCATGCATGTGCTGGCACCATCGGGCACCTTCGTCCCCACGACGCTGGACACCGACTTCCTGCTGTTGGCCGCGGGCAGCGGGATCACCCCGATGATGGCGATCTGTAAGTCCGCCCTCGCCGAGGGCTCCGGAAAGGTCGTCCTGGTCTACGCCAACCGTGACGAGAACTCGGTCATCTTCGCCGATGCGCTGCGTGAGTTGGCCGCCGCGCACCCGGACCGGCTGACGGTGATCCACTGGCTGGAGACCGTACAGGGGTTGCCCAACACCGATGCGCTGGCGACGCTGGTGCGACCGTTCGCCGCATATGAGGCATTCATCTGCGGGCCCGGCCCGTTCATGTCCGCCGCCGAAGCGGCGTGCAAGACCGTCGATGCCAGGCATATCCACATCGAGGTGTTCAAGTCGCTGGATTCCGACCCGTTCGCTCAGGTCGTCATAGACGTGGACGAAGACGACGACCGCGGCCCCGCGCAGGCGATCGTCGAACTCGACGGCACCACCCACGAGATCGAATGGCCGCGTAAGGCCAAGTTGCTCGATGTGCTGCTGAACAAGGGTCTGGACGCACCGTTCTCCTGCCGGGAGGGCCACTGCGGGGCGTGCGCGGTGCTGATGCGCAAGGGCGATGTCGAGATGGAGATCAACGATGTCCTGGAGCCGTCCGATCTCGACGAGGGCCTCATCCTGGCCTGCCAGGCTTTGCCGACATCGGATTCGGTGGAAGTCACCTACGACGAATAG |

**Table S3. Primers for constructing knockout vectors**

| **Targeted gene** | **Primers** | **Sequence** | **Note** |
| --- | --- | --- | --- |
| *hsd4A1* | Hsd4A1-D-F | ATAAGAATGCGGCCGCTCTGCGAGAACGTCATTT | *Not* Ⅰ |
|  | Hsd4A1-D-R | CCCAAGCTTGATGCCAGCACACGCGGC | Hind Ⅲ |
|  | Hsd4A1-U-F | GAAGATCTTGCCGAAATGCATGATCG | *Bgl* Ⅱ |
|  | Hsd4A1-U-R | ATAGTTTAGCGGCCGCCGCTGCTTCAGGGGACAC | *Not* Ⅰ |
| *hsd4A2* | Hsd4A2-D-F | GGAATTCGCCAAGTCGCGCACCCCGCT | *EcoR* Ⅰ |
|  | Hsd4A2-D-R | CCCAAGCTTATCCGTGCTCGTTTCGACGT | *Hind* Ⅲ |
|  | Hsd4A2-U-F | CGGGATCCCAGGATCACCACGTGGGTGC | *BamH* Ⅰ |
|  | Hsd4A2-U-R | GGAATTCCGTCACGATGACTACCCGGC | *EcoR* Ⅰ |
| *fadA5* | FadA5-D-F | ATGTGCGCCGGTGGCGCGCTGAGCTCTAGAGC | *Xba* Ⅰ |
|  | FadA5-D-R | CCAAGCTTACAGGCACATCAACCACAGGAGTGC | *Hind* Ⅲ |
|  | FadA5-U-F | CGGGATCCGCTCCCACTGGTCGGGATTGTCGGCGAAGG | *BamH* Ⅰ |
|  | FadA5-U-R | GCTCTAGAGCGCTTGCCGATGGGGCTGCGGG | *Xba* Ⅰ |

**Table S4. Primers used for gene overexpression**

| **Overexpressed genes** | **Primers** | **Sequence** |
| --- | --- | --- |
| *kshA395* | KshA395-F | GGAATTCCATATG ACCGATATCCGCGAG |
|  | KshA395-R | GGAATTC TCACCGTTGCGCGGTGGT |
| *kshB122* | KshB122-F | GGAATTCGTGACGGAGGAACCGCTC |
|  | KshB122-R | CCCAAGCTTCTATTCGTCGTAGGTGAC |
| *kstd2* | KstD2-F | GGAATTCATGACTGAACAGGACTACAG |
|  | KstD2-R | GGAATTCTCAGGCCTTTCCAGCGAG |

**Table S5. Summary of** **gene complementation**

| **Starting strain** | **KI Gene** | **Complemented strain** | **Regain of function** |
| --- | --- | --- | --- |
| HGMS6*^ΔkshA226^* | *kshA226* | HGMS6*^ΔkshA226/kshA226/^* | YES |
| HGMS6*^ΔkshA226/ΔopccR667^* | *opccR667* | HGMS6*^ΔkshA226/ΔOpccR667/opccR667^* | YES |
| HGMS6*^Δhsd4A1^* | *hsd4A1* | HGMS6*^Δhsd4A1/hsd4A1^* | YES |
| HGMS6*^Δhsd4A1/Δhsd4A2^* | *hsd4A2* | HGMS6*^Δhsd4A1/hsd4A2/ hsd4A2^* | YES |
| HGMS2*^Δhsd4A1^* | *hsd4A1* | HGMS2*^Δhsd4A1/ hsd4A1^* | YES |
| HGMS2*^Δhsd4A2^* | *hsd4A2* | HGMS2*^Δhsd4A2/ hsd4A2^* | YES |
| HGMS2*^ΔfadA5^* | *fadA5* | HGMS2*^ΔfadA5/ fadA5^* | YES |
| HGMS2*^Δkstd1/Δhsd4A1^* | *hsd4A1* | HGMS2*^Δkstd1/Δhsd4A1/ hsd4A1^* | YES |
| HGMS2*^Δkstd1/Δhsd4A2^* | *hsd4A2* | HGMS2*^Δkstd1/Δhsd4A2/ hsd4A2^* | YES |
| HGMS2*^Δkstd1/ΔfadA5^* | *fadA5* | HGMS2*^Δkstd1/ΔfadA5/ fadA5^* | YES |


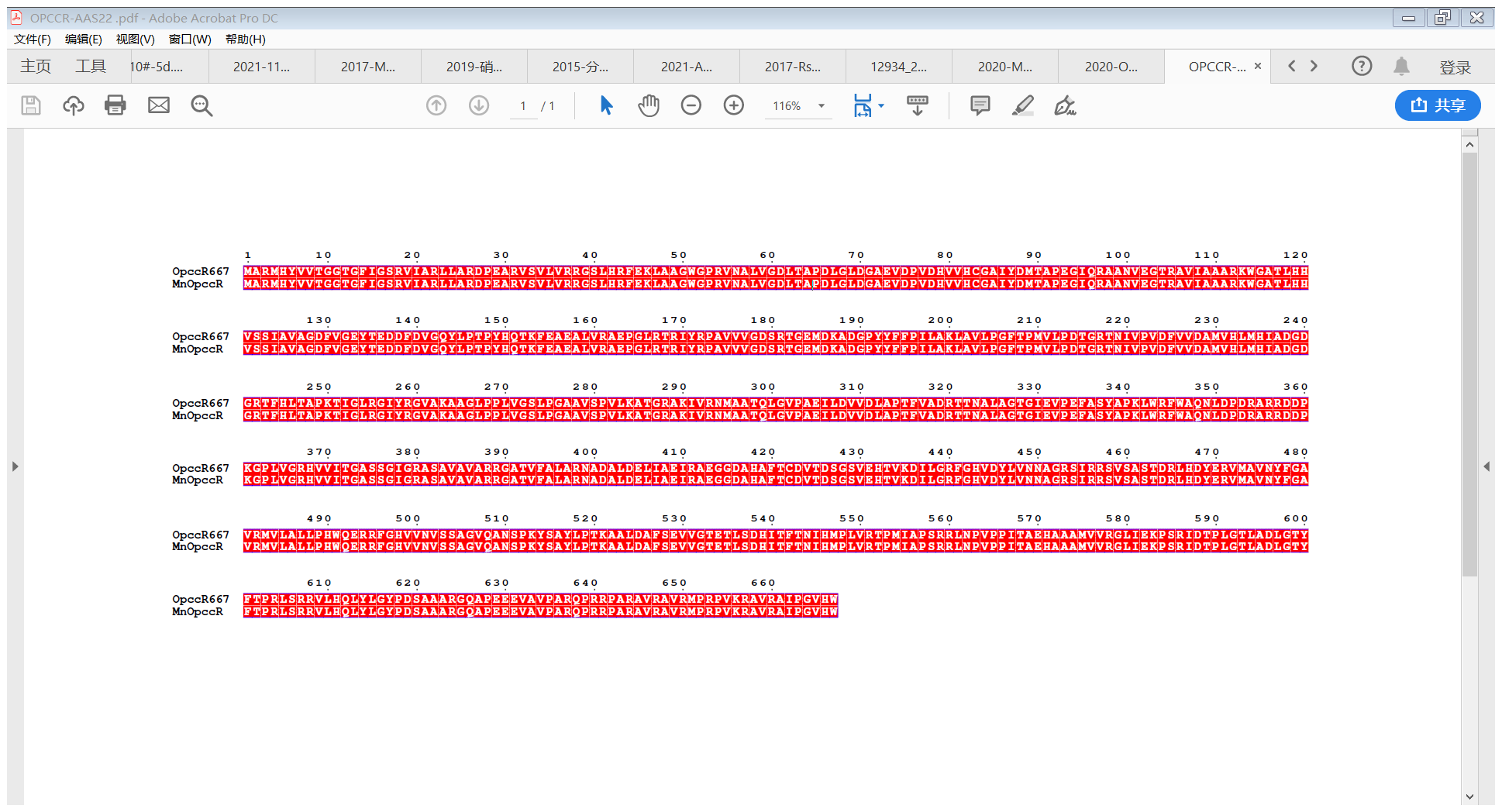


**Fig. S1. Amino acid alignment of OpccR667 with MnOpccR enzyme.** MnOpccR: *Mycobacterium sp*. CCTCC AB2019054

~~
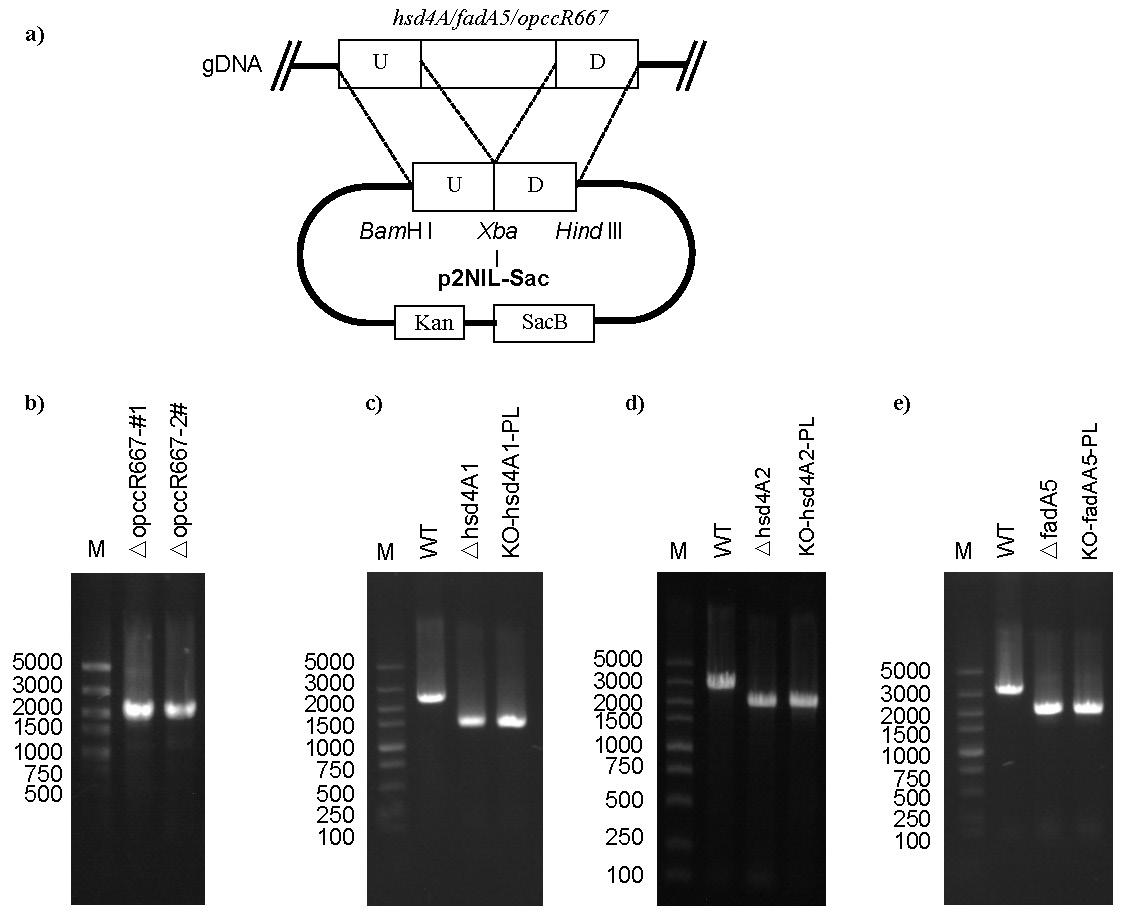
~~

**Fig. S2. Generation of the *opccR667*-, *hsdA1-, hsdA2-* and *fadA5-*deficient mutants. a**. Schematic diagram of the homologous recombination vector. U: the upstream sequence; D: the downstream sequence. **b**. PCR confirmation of putative *opccR667*-knockout colonies. ΔopccR667-#1 and ΔopccR667-#1 were two different colonies. **c**. PCR confirmation of putative *hsdA1*-knockout colony. WT: HGMS2; Δhsd4A1: putative Δhsd4A1-default colony; KO-hsd4A1-PL: recombination plasmid for knocking *hsd4A1* out. **d**. PCR confirmation of putative *hsd4A2*knockout colony. WT: HGMS2; Δhsd4A2: putative Δhsd4A2-default colony; KO-hsd4A2-PL: recombination plasmid for knocking *hsd4A2* out. **e**. PCR confirmation of putative *fadA5*-knockout colony. WT: HGMS2; ΔfadA5: putative ΔfadA5-default colony; KO-fadA5-PL: recombination plasmid for knocking *fadA5* out.


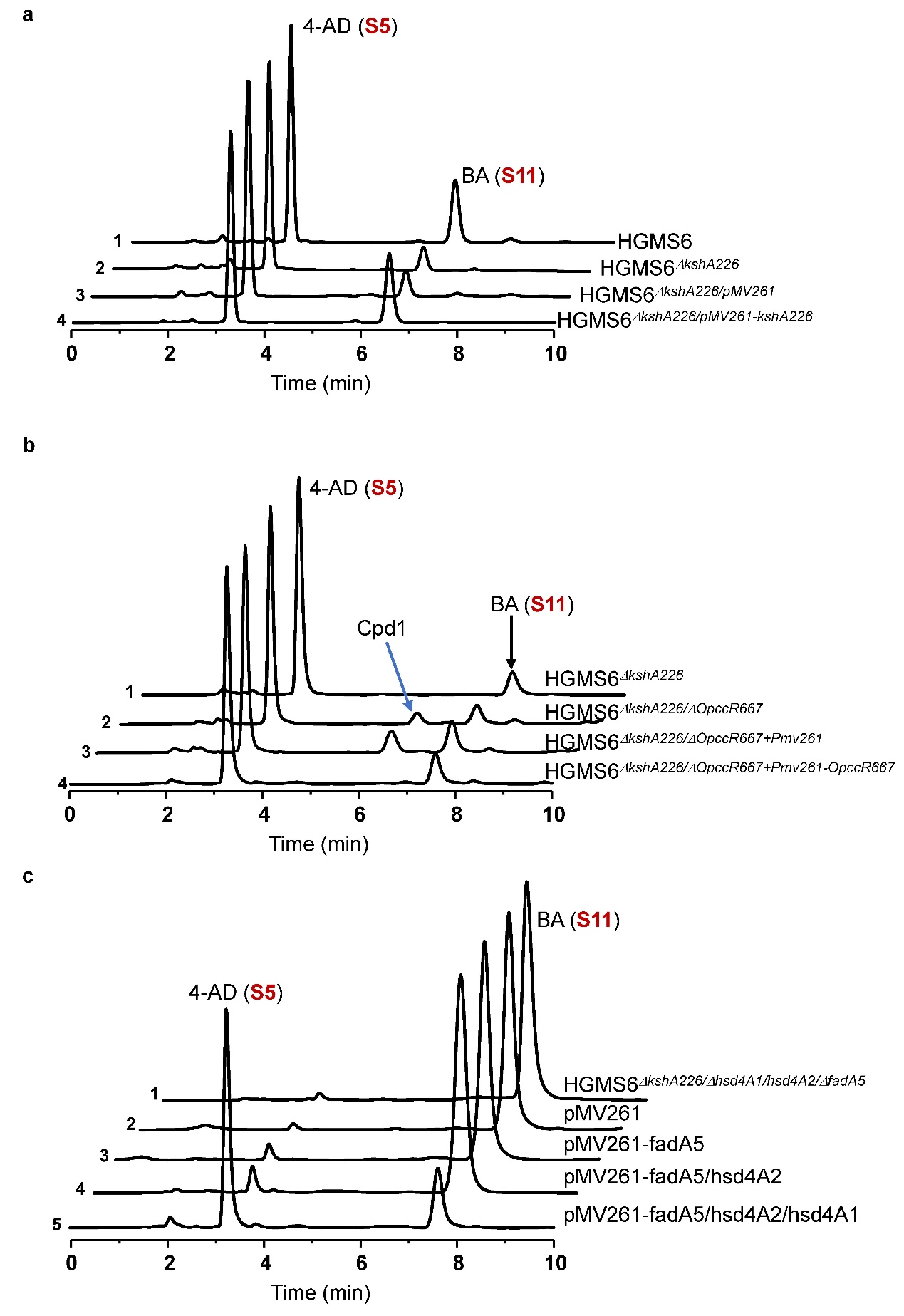


**Fig. S3.**  **The complementation assays of the deleted *OpccR667, kshA226, hsd4A* and *fadA5* genes by homologous expression. a**) HPLC assay of the phytosterol conversions by the HGMS6*^ΔkshA226^* with complementation. 1. HGMS6; 2. HGMS6*^ΔkshA226^;* 3. HGMS6*^ΔkshA226+pMV261^*; 4. HGMS6*^ΔkshA226+pMV261-kshA226^*. **b**) HPLC assay of the phytosterol conversions by the HGMS6*^ΔkshA226/ΔOpccR667^* with complementation. 1. HGMS6*^ΔkshA226^*; 2. HGMS6*^ΔkshA226/ΔOpccR667^*; 3. HGMS6*^ΔkshA226/ΔOpccR667+pMV261^*; 4. HGMS7*^ΔOpccR667+^*^pMV261-OpccR667^. **c**) HPLC assay of the phytosterol conversions by the HGMS6*^ΔkshA226/Δhsd4A1/hsd4A2/ΔfadA5^* with complementation. 1. HGMS6*^ΔkshA226/Δhsd4A1/hsd4A2/ΔfadA5^*; 2. HGMS6*^ΔkshA226/Δhsd4A1/hsd4A2/ΔfadA5+pMV261^*; 3. HGMS6*^ΔkshA226/Δhsd4A1/hsd4A2/ΔfadA5+pMV261-fadA5^*; 4. HGMS6*^ΔkshA226/Δhsd4A1/hsd4A2/ΔfadA5+pMV261-fadA5/hsd4A2^*; 5. HGMS6*^ΔkshA226/Δhsd4A1/hsd4A2/ΔfadA5+pMV261-fadA5/hsd4A2/hsd4A1^*.


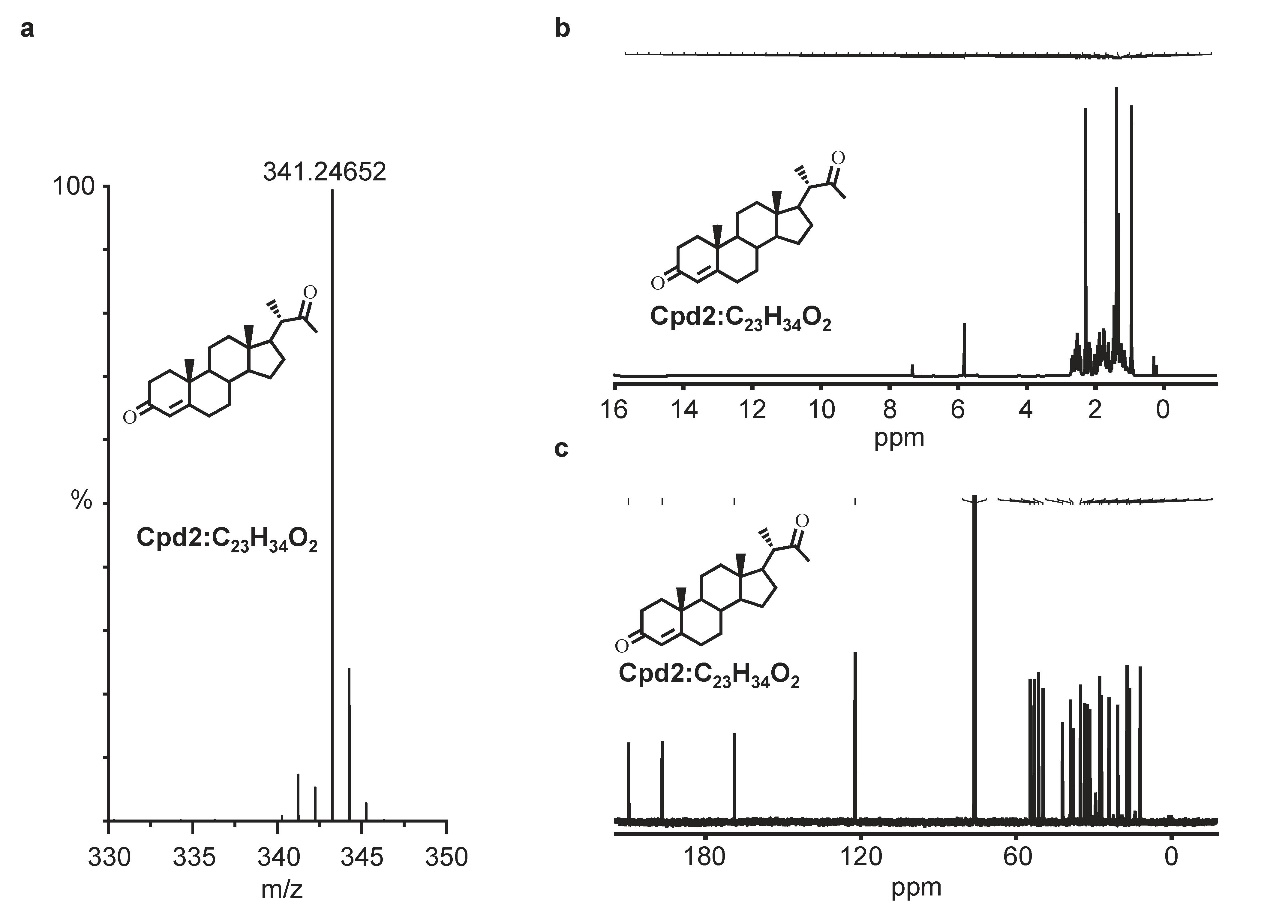


**Fig. S4. Structure characterization of Cpd2. a**). Mass spectrum of Cpd2. **b**). ^1^H NMR spectrum of Cpd2. **c**). 13C NMR spectrum of Cpd2.


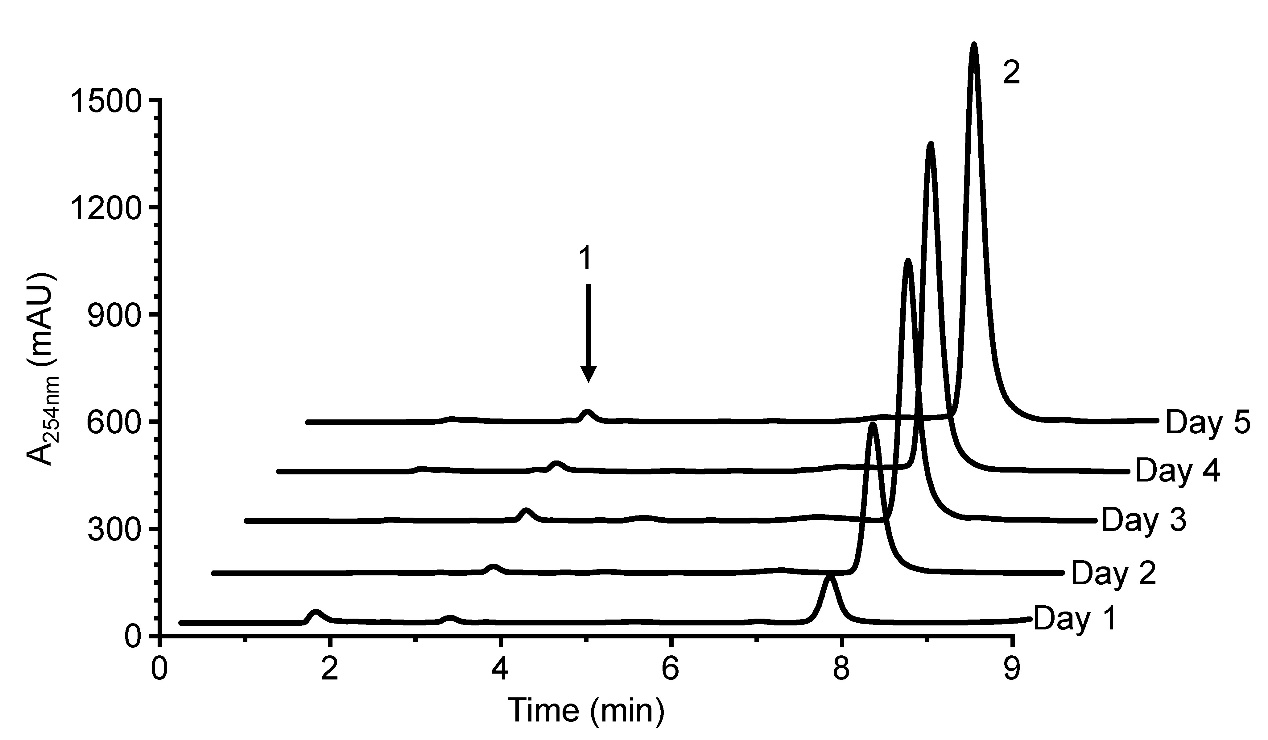


**Fig. S5. HPLC profiles of samples extracted from the fermentation broth during 5-day fermentation.** 1. 4-AD; 2. BA.
